# Chromeno[3,4-*b*]xanthones as First-in-Class AChE and Aβ Aggregation Dual-Inhibitors

**DOI:** 10.3390/ijms22084145

**Published:** 2021-04-16

**Authors:** Daniela Malafaia, Ana Oliveira, Pedro A. Fernandes, Maria J. Ramos, Hélio M. T. Albuquerque, Artur M. S. Silva

**Affiliations:** 1 LAQV-REQUIMTE, Department of Chemistry, University of Aveiro, Campus de Santiago, 3810-193 Aveiro, Portugal; danielamalafaia@ua.pt; 2 LAQV-REQUIMTE, Computational Biochemistry Laboratory, Department of Chemistry and Biochemistry, Faculty of Sciences, University of Porto, Rua do Campo Alegre, s/n, 4169-007 Porto, Portugal; anoliveira@fc.up.pt (A.O.); pafernan@fc.up.pt (P.A.F.); mjramos@fc.up.pt (M.J.R.)

**Keywords:** Alzheimer’s disease, AChE, Aβ-aggregation, dual-target inhibitors, chromeno[3,4-*b*]xanthones

## Abstract

Alzheimer’s disease (AD) is a complex multifactorial disorder, mainly characterized by the progressive loss of memory and cognitive, motor, and functional capacity. The absence of effective therapies available for AD alongside the consecutive failures in the central nervous system (CNS) drug development has been motivating the search for new disease-modifying therapeutic strategies for this disease. To address this issue, the multitarget directed ligands (MTDLs) are emerging as a therapeutic alternative to target the multiple AD-related factors. Following this concept, herein we describe the design, synthesis, and biological evaluation of a family of chromeno[3,4-*b*]xanthones as well as their (*E*)-2-[2-(propargyloxy)styryl]chromone precursors, as first-in-class acetylcholinesterase (AChE) and β-amyloid (Aβ) aggregation dual-inhibitors. Compounds **4b** and **10** emerged as well-balanced dual-target inhibitors, with IC_50_ values of 3.9 and 2.9 μM for AChE and inhibitory percentages of 70 and 66% for Aβ aggregation, respectively. The molecular docking showed that most of the compounds bound to AChE through hydrogen bonds with residues of the catalytic triad and π-stacking interactions between the main scaffold and the aromatic residues present in the binding pocket. The interesting well-balanced activities of these compounds makes them interesting templates for the development of new multitarget compounds for AD.

## 1. Introduction

Alzheimer’s disease (AD) is the most common form of dementia and contributes to 60–70% of all cases, affecting especially the elderly population, with ages above 65 [1]. This neurodegenerative disorder is mainly characterized by the neurodegeneration of several areas of the brain, particularly in the cerebral cortex and hippocampus, which leads to the progressive and unremitting loss of memory and cognitive, motor, and functional capacity [2,3]. Presently, the clinical diagnosis of AD is mainly based on mental and cognitive evaluation and its pathological confirmation is only achieved through the post-mortem histopathological examination of brain tissue [4].

Despite many efforts from the scientific community, the exact cause of AD remains unclear; however, it has been stated that several hypothesis are associated with the pathological development of the disease, including acetylcholinesterase (AChE) and amyloid-β (Aβ) [5,6]. AChE is the enzyme responsible for the hydrolysis and inactivation of the neurotransmitter acetylcholine (ACh) and subsequent regulation of its concentration in the synaptic cleft [7]. The ACh-mediated neurotransmission is crucial for the nervous system function since its blockade and/or loss is correlated with the progressive deterioration of cognitive, autonomic, and neuromuscular functions, as in AD. Over the years, several studies have been reporting that lower levels of ACh are straightly correlated with AChE activity and, thus, AChE is considered one of the main targets for AD therapy. In 1990s cholinesterase inhibitors were introduced to the market as the first therapeutics against AD. These drugs increase acetylcholine levels in the brain by inhibiting AChE; however, they offer only time-limited symptomatic relief for the patients. Nevertheless, cholinesterases are still considered important biological targets in the design of multifunctional ligands. Usually, the inhibitory activity against cholinesterases is combined with the activity towards disease-modifying targets such as Aβ or tau aggregation [2,8,9].

On the other hand, the Aβ-peptides are generated by the proteolysis of its amyloid precursor protein (APP) [10,11,12]. Once in the cell surface, this protein can be cleaved at its *N*-terminus by β-secretase, leading to the production of the soluble amyloid precursor protein β-fragment (APPsβ) and the membrane-bound *C*-terminus, C99 [10,13,14]. This remaining fragment is then cleaved by γ-secretase, which generates Aβ-peptides [13,15,16]. These peptides are characterized by a long, usually ribbon-like morphology and share a common “cross-β” structure, at a molecular level, which consists of laminated β-sheets [17,18]. The accumulation of Aβ-peptides leads to the production of insoluble fibrils, which ultimately aggregate into the characteristic Aβ plaques of AD [15].

The current clinical therapy for AD is mainly divided in acetylcholinesterase inhibitors (AChEIs), represented by Donepezil, Galantamine, Rivastigmine, and Tacrine, which has already been discontinued due to its hepatoxicity, and the antagonist of the *N*-methyl-D-aspartic acid (NMDA) receptor, Memantine, all approved by the US Food and Drug Administration (FDA) and European Medicines Agency (EMA) [1,5]. This approach only provides symptomatic relief at short-term and do not decreases the disease progression, leading to the damage of the neurons and ultimately to the patient’s death [5].

The relationship between the histopathological hallmarks of AD, i.e., Aβ plaques, neurofibrillary tangles (NFTs) and AD-associated cognitive impairment has been strengthening its multifactorial nature and may help to explain the consecutive failures in the disease-modifying drug discovery [19]. To address this issue, the multitarget directed ligands (MTDLs) have been emerging as an alternative approach to focus on the multiple AD-related factors [20,21]. This strategy is based on the simultaneous molecular recognition of multiple targets associated with the same pathology by a single molecule, which combines different pharmacophores of different bioactive molecules [22,23].

**Figure 1:**
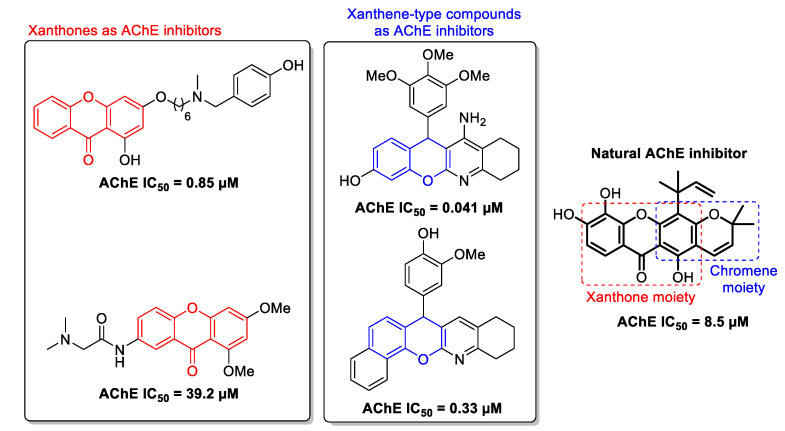
Examples of xanthones and xanthene-type compounds as AChE inhibitors.

Natural products often play important roles in drug discovery and development processes. Xanthones are important examples of that, presenting a wide range of biological activities, particularly as AChE inhibitors (Figure 1) [24,25]. Furthermore, several xanthone derivatives have been reported for their dual/multitarget activity as potential agents to treat AD [26]. Alongside xanthones, chromenes are also important pharmacophores, reported to have AChE inhibition properties (Figure 1) [27]. An interesting example is the natural macluraxanthone (Figure 1), which contains a chromene nucleus naturally overlapped to a xanthone scaffold, presenting good AChE inhibition properties [28].

**Figure 2:**
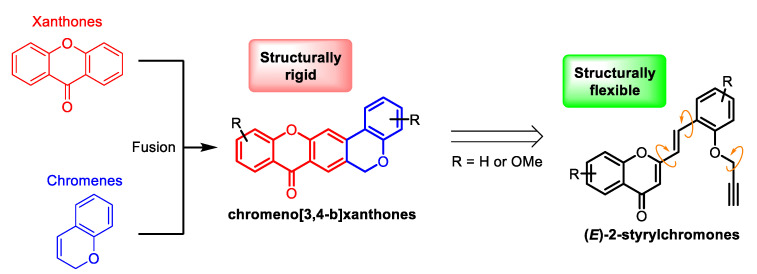
Retrosynthetic design strategy of chromeno[3,4-*b*]xanthones and their structurally flexible precursors.

Inspired by the above examples and following the MTDLs concept, in this work, we describe the design, synthesis, and biological evaluation of a series of chromeno[3,4-*b*]xanthones and their structurally flexible precursors (*E*)-2-[2-(propargyloxy)styryl]chromones (Figure 2), as first-in-class dual-inhibitors of both AChE and Aβ aggregation.

The target compounds—chromeno[3,4-*b*]xanthones—feature xanthone and chromene pharmacophoric units, fused to each other in one new molecular entity, expected to have enhanced anticholinesterase potency (Figure 2). Their (*E*)-2-styrylchromone precursors were also evaluated against both targets, to establish structure-activity relationships (SAR) and to get insights whether structurally flexible or rigid chromone-derived compounds are better templates for the development of AChE and Aβ aggregation dual-inhibitors. Beyond that, the hybrid structure of chromeno[3,4-*b*]xanthones represent highly aromatic/hydrophobic flat extended frameworks, which is a common feature of the existing Aβ aggregation inhibitors, together with hydrogen bonding spots such as methoxy or hydroxy groups (Figure 2), and therefore they can also be effective against Aβ fibrillation [29].

## 2. Results and Discussion

### 2.1. Synthesis of Target Compounds

**Scheme 1:**
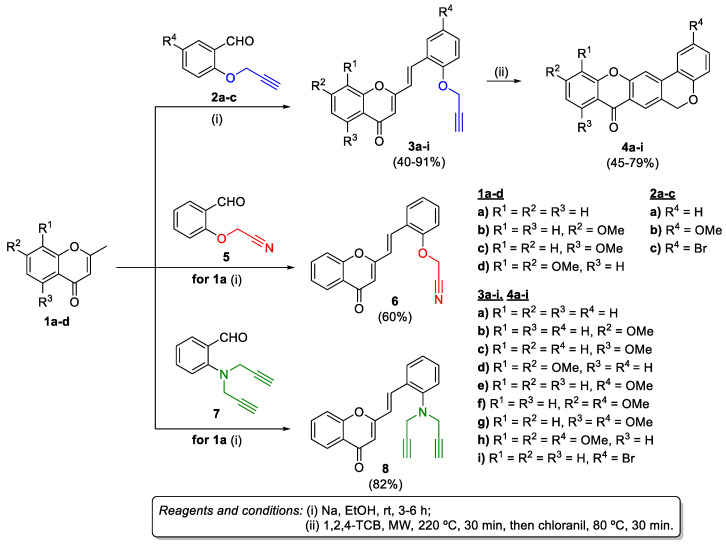
Synthesis of chromeno[3,4-*b*]xanthones **4a-i**, and (*E*)-2-styrylchromones **3a-i**, **6**, and 8. 1,2,4-Trichlorobenzene (1,2,4-TCB).

The synthetic strategy for chromeno[3,4-*b*]xanthones **4a-i** encompasses two reaction steps (Scheme 1). First, the base-promoted aldol reaction between 2-methylchromones **1a-d** and aldehydes **2a-c**, gave the intermediate (*E*)-2-styrylchromones **3a-i** in 40–91% yield (Scheme 1). The (*E*)-2-styrylchromones **3a-i** were subsequently used as substrates in MW-assisted intramolecular Diels-Alder (DA) reactions, allowing the preparation of the target chromeno[3,4-*b*]xanthones **4a-i** in 45–79% yield (Scheme 1). 

The (*E*)-2-styrylchromone nitrogen-containing analogues **6** and **8**, were prepared in good yields (60% and 82%, respectively), following the same reaction conditions as described for derivatives **3a-i**, using the 2-methylchromone **1a** and aldehydes **5** and **7**, respectively (Scheme 1). The 2-methylchromones **1a-d** as well as the aldehydes **2a-c**, **5** and **7** are commercially unavailable, and therefore they were prepared following reported methodologies (see SI for full details, Schemes S1–S3).

**Scheme 2:**
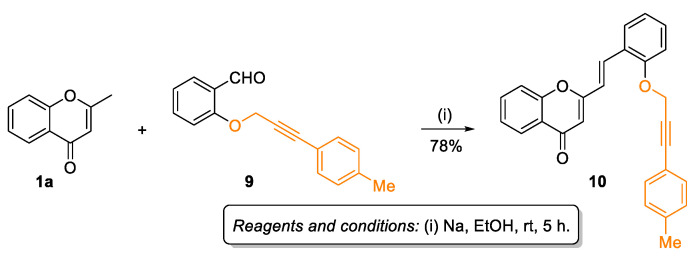
Synthesis of (*E*)-2-styrylchromone **10**.

The (*E*)-2-styrylchromone **10**, with an additional aryl ring substituting the terminal alkyne, was also prepared in 78% yield from the aldol reaction of 2-methylchromone **1a** and aldehyde **9** (Scheme 2). Aldehyde **9** was obtained through Sonogashira cross-coupling reaction of 2-(propargyloxy)benzaldehyde **2a** and 1-iodo-4-methylbenzene (see SI for full details, Scheme S4).

### 2.2. Biological Evaluation

All the synthesized chromeno[3,4-*b*]xanthones **4a-i**, as well as their precursors (*E*)-2′-propargyloxy-2-styrylchromones **3a-i** were evaluated in vitro for their potential as dual-target agents, against the selected AD targets—AChE and Aβ aggregation. Both nitrogen-containing analogues **6** and **8**, as well as (*E*)-2-styrylchromone **10** were also evaluated to explore the possibility to prepare more efficient structural analogues of chromeno[3,4-*b*]xanthones **4a-i**. Molecular modelling studies were conducted to explain the enzyme-inhibitors interaction and to support the SAR analysis discussion. 

#### 2.2.1. AChE Inhibition

The synthesized compounds were evaluated for their inhibitory activity against AChE (*Electrophorus electricus—ee*AChE), using an adaptation of Ellman’s method [30]. The *ee*AChE is an excellent model system as this enzyme and the human enzyme display a remarkably extensive sequence identity (>85%). Initially, we performed a screening assay for the synthesized compounds to determine their enzyme inhibition at 20 μΜ as a threshold maximum concentration. Based on this preliminary study, the compounds with inhibitory potency higher than 50% were established, for which the IC50 values were determined. The obtained results from the in vitro assays are represented in Table 1. Donepezil was used as reference compound.

Among the 21 tested compounds, eight displayed interesting anticholinesterase profile, with IC_50_ values ranging from 2.1 to 9.5 μΜ (Table 1), just two orders of magnitude higher than Donepezil. Given that the inhibition constant of Donepezil for AChE is in the low-nanomolar range, the IC_50_ of some of the tested compounds is remarkable, in particular because they have not been optimized yet.

**Figure 3:**
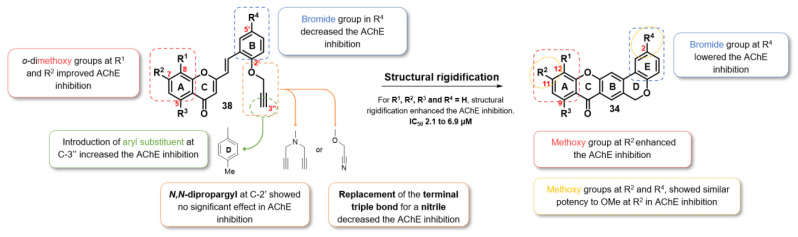
SAR profile towards *ee*AChE of (*E*)-2-styrylchromones **3a-i**, **6**, **8** and **10** and chromeno[3,4-*b*]xanthones **4a-i**.

The obtained results showed that the most active compounds were the (*E*)-2-styrylchromones **3d** and **10**, and the chromeno[3,4-*b*]xanthones **4a**, **4b**, **4e**, and **4f** (highlighted in blue, Table 1). The first SAR analysis to the (*E*)-2-styrylchromones **3a-i** revealed that the overall introduction of methoxy groups throughout the main core decreased their anticholinesterase activity, when compared to the unsubstituted derivative **3a** (Table 1, Figure 3), except for derivative **3d**, in which the presence of *ortho*-dimethoxy group (donepezil fragment) in A-ring increased its anticholinesterase activity (Table 1, Figure 3). The (*E*)-2-styrylchromone **10** also displayed interesting inhibitory activity against AChE, which seems to indicate that the addition of the D-ring may be advantageous for AChE activity in comparison with the parent compounds **3a-i** (Table 1, Figure 3). The replacement of the terminal triple bond (compound **3a**) for a nitrile group in compound **6** decreased the AChE activity, suggesting the triple bond as an essential pharmacophoric fragment. Finally, the replacement of the *O*-propargyl group for the *N,N*-dipropargyl moiety in compound **8** did not show meaningful difference in AChE activity, when compared to the parent compound **3a** (Table 1, Figure 3).

Conversely, the rigid structure of chromeno[3,4-*b*]xanthones **4a-i** showed better overall results, when compared to the respective (*E*)-2-styrylchromone precursors **3a-i** (Table 1). The unsubstituted chromeno[3,4-*b*]xanthone **4a** exhibited the lowest IC_50_ of the series, being the introduction of methoxy groups disadvantageous for AChE activity, mainly for derivatives **4c**, **4d**, **4g** and **4h** (Table 1, Figure 3). The introduction of Br at C-4′ and C-3 of derivatives **3i** and **4i**, respectively, decreased their inhibitory potential against AChE, when compared to their respective unsubstituted analogues **3a** and **4a** (Table 1, Figure 3).

Generically, the presence of methoxy groups in chromeno[3,4-*b*]xanthones **4a-h** seems to be more important for AChE activity than in their precursors **3a-h**. On the other hand, chromeno[3,4-*b*]xanthones **4a-i** can be understood as a structural rigidification of their precursors (*E*)-2′-propargyloxy-2-styrylchromones **3a-i**. In this way, the obtained inhibition potencies clearly show that the rigid structures of chromeno[3,4-*b*]xanthones **4a-i** are more effective AChE inhibitors in detriment to the flexible structures of their precursors (Table 1, Figure 3).

#### 2.2.2. Molecular Docking

To understand the structural basis of the AChE inhibitory activity determined for (*E*)-2′-styrylchromones **3a-i**, **6**, and **10** as well as for the chromeno[3,4-*b*]xanthones **4a-i**, the binding mode to AChE was studied, using the GOLD software [31].

First, the accuracy of the protocol used in the molecular docking was validated. To do so, we re-docked the huprine ligand co-crystallized in the human acetylcholinesterase (hAChE) and the results indicated that the docking algorithm perfectly reproduced the x-ray structure of the hAChE:huprine complex. Subsequently we docked the huprine ligand into the *ee*AChE structure and observed that the binding modes are comparable in the two organisms. The two enzymes are very similar, sharing as much as >85% sequence identity, and display fully overlapping binding pockets. Thus, it is not surprising that the docking algorithm would behave similarly in both cases. We concluded that it was suitable to proceed with the analysis of the chosen compounds (see SI, Figures S1 and S2).

**Figure 4:**
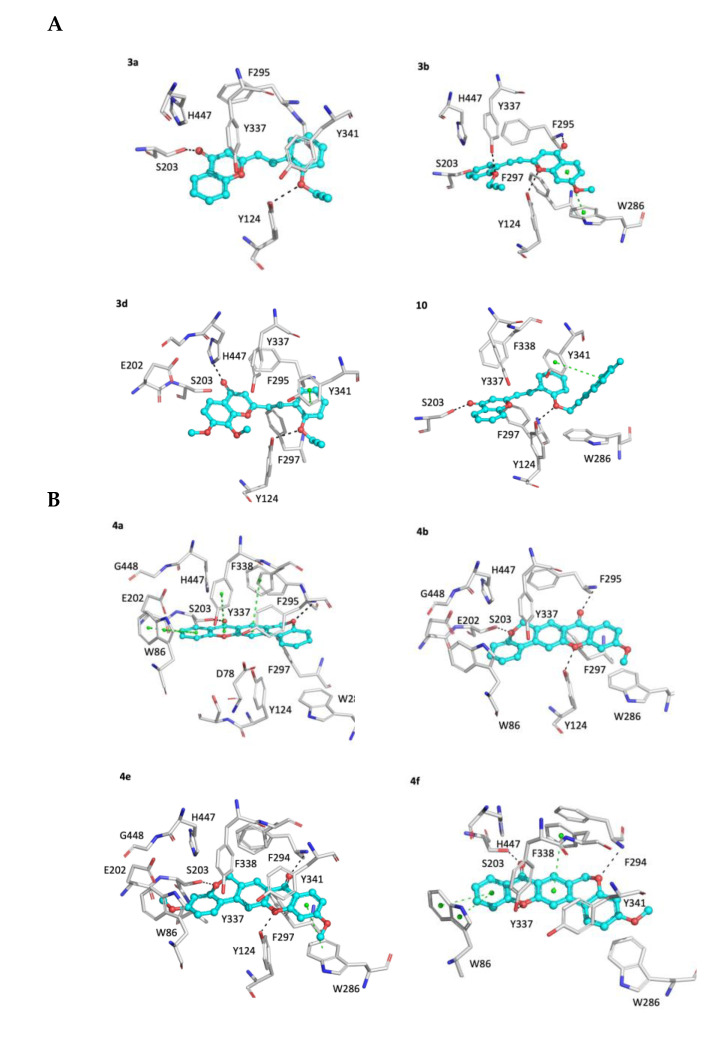
Summary of the docking calculation results, and indication of the binding poses adopted by: (**A**) (*E*)-2-styrylchromones **3a**, **3b**, **3d** and **10** and (**B**) chromeno[3,4-*b*]xanthones **4a**, **4b**, **4e**, and **4f**. The hydrogen bonds are showed as black dash lines and the π-stacking interactions in green dash lines.

To determine the substituents importance for the binding, the docking results were visually inspected by dividing the compound series in agreement with their main scaffold. First, it was studied the pattern of interactions observed for (*E*)-2-styrylchromones **3a-i**, **6**, and 10, and a few examples are illustrated in Figure 4A. Here, it was observed that either Ser203 or His443 formed a hydrogen bond with the ether moiety in most of the compounds, except for compounds **3b**, **3e**, and 3f. Both Ser203 and His443 are key residues of the catalytic triad and are implicated on the hydrolysis of the carboxyl ester during the enzymatic mechanism. Moreover, the ether substituent in the (*E*)-2′-propargyloxy-2-styrylchromones **3** was stabilized by the Tyr124 side chain and by a π-stacking interaction either with Trp286 or with Tyr341. The compounds that showed the best inhibition were **3d** and **10**, both forming a perfect stacking with the Tyr341 side chain. Compound **3b** was unable to perform this interaction and stacked with Trp286 instead. Compound **3e** tried to bind in a similar position to **3d** but the results implied that the methoxy group at **3e** was destabilized by the vicinity with Glu202. This may justify why this resulted in a weaker inhibition of the enzyme (Figure 4A).

Finally, the docking results obtained for chromeno[3,4-*b*]xanthones **4a-i** reinforced, once again, the role of Ser203, and also Phe294, which formed hydrogen bonding interactions with the heterocyclic rings, in all the studied compounds, except for compound **4e**, which formed an additional hydrogen bond with the adjacent residue His443. The chromeno[3,4-*b*]xanthones **4a-i** binding was also stabilized by several π-stacking interactions between the main scaffold and aromatic residues present in the binding pocket Trp86, Trp286, Phe338, and Tyr341. Moreover, the results did not show any specific interactions between the enzyme and the methoxy groups. However, there was a preferential trend to accommodate the methoxy group towards the region occupied by Trp286. Only when two methoxy substituents are present, the second group occupies the anionic subsite of the enzyme, surrounded by Glu202, Trp86 and Tyr337 (Figure 4B). The enzyme can accommodate one or two methoxy groups in the binding pocket but only if the substituents do not occupy *orto*-positions; otherwise, it leads to steric clashes. The presence of the bromide group in compound **4i** also destabilized the binding as it led to electrostatic repulsions. The experimental binding affinity, alongside the molecular docking results, suggested that there is a similar probability for the compounds to bind in two equivalent positions, related by a 180° flip around the ligand center. The small differences in the binding affinity are probably due to the cost of the methoxy accommodation, resulting in small re-adjustments of the residues of the binding pocket. This explains why compound **4a**, which perfectly formed three π-stacking interactions and two hydrogen bonds, was the best compound exploited among all. 

#### 2.2.3. Aβ Aggregation Inhibition

**Figure 5:**
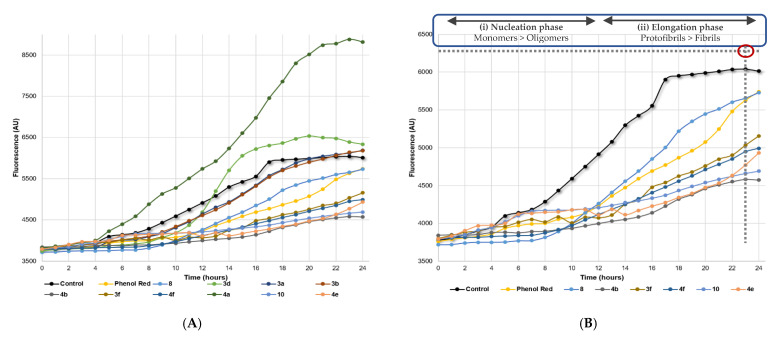
ThT-Aβ aggregation assay with (**A**) (*E*)-2-styrylchromones **3a**, **3b**, **3d**, **3f**, **8** and **10**, and chromeno[3,4-*b*]xanthones **4a**, **4b**, **4e**, and **4f** and (**B**) (*E*)-2-styrylchromones **3f**, **8** and **10** and chromeno[3,4-*b*]xanthones **4b**, **4e**, and **4f**, monitored by ThT fluorescence.

The formation of amyloid aggregates is crucial for the path mechanism of AD. Therefore, we also decided to check the inhibitory activity of the synthesized compounds against the Aβ aggregation. To do so, we conducted a thioflavin T (ThT)-based fluorimetric assay to evaluate their ability to inhibit β-amyloid aggregation in vitro, using 1,1,1,3,3,3,-hexafluoro-2-propanol (HFIP)-treated Aβ1-42 peptides. This assay is based on the interaction between benzothiazole dye (ThT) and cross β-sheet quaternary structure of the amyloid protein. The intensity of fluorescence is weak when the dye is free and increases when the dye binds to β-sheet structures [32]. Cumulative evidence has been shown that aromatic/hydrophobic areas and hydrogen bonding components (such as hydroxy or methoxy groups) along flat extended structures in small-molecule inhibitors [18,29] as in chromeno[3,4-*b*]xanthones **4a-i**, are important structural features for Aβ aggregation inhibitors. However, in line with objectives of the present work and encouraged by the obtained results so far, we selected the best compounds from the AChE inhibition assay and performed an in vitro inhibition assay at 20 μM, to evaluate the compounds inhibition potencies against Aβ aggregation and their subsequent dual-target profile. The ThT fluorescence data was recorded over 24 h and the obtained results were then plotted against time, as illustrated in Figure 5. Phenol red was used as the reference inhibitor for in vitro Aβ aggregation. Almost all the tested compounds were found to have good antiaggregating activity when compared to control [ThT, Aβ and dimethylsulfoxide (DMSO) as control vehicle] as well as to the reference compound, except for chromeno[3,4-*b*]xanthone **4a**, and (*E*)-2-styrylchromones **3a**, **3b**, and **3d**, as depicted in Figure 5A.

In a more detailed analysis, we highlighted the compounds with the best results in Figure 5B. The amyloid fibrillation comprises two main stages: (i) the nucleation phase, in which the monomers undergo a conformational change and associate to form oligomers, and (ii) the elongation phase, in which the oligomers rapidly grow by further addition of more monomers and form larger fibrils, until saturation (blue box, Figure 5B) [33]. As shown in Figure 5B, the Aβ_1-42_ peptides alone were completely aggregated after 20–24 h and consequently the ThT fluorescence intensity reached its highest value, while the treatment with the assay compounds resulted in a delay of the nucleation phase of the Aβ formation and, subsequently, in a decrease of the fluorescence intensity. Moreover, almost all compounds showed an even bigger delay in the nucleation phase (except for compound **8**), when compared to the reference compound, Phenol Red.

The analysis of the averaged fluorescence intensities at the plateau showed that compounds **3f**, **8**, **10**, **4b**, **4e**, and **4f** inhibited the Aβ aggregation in a range of 18–70%, even though the rest of them turned out to be weak inhibitors (Table 2). The best results were obtained for chromeno[3,4-*b*]xanthones **4b**, **4e**, **4f**, and (*E*)-2-styrylchromone **10**, particularly the first one with an inhibition percentage of 70% (highlighted in blue, Table 2).

**Figure 6:**
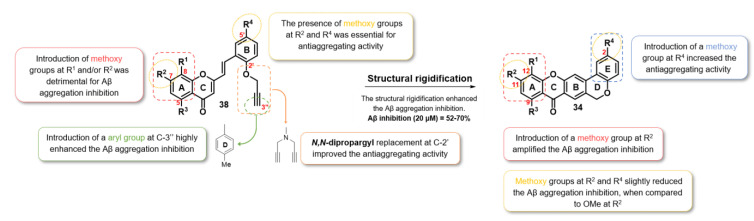
SAR profile towards Aβ aggregation of (*E*)-2′-styrylchromones **3a**, **3b**, **3d**, **3f**, **8** and **10** and chromeno[3,4-*b*]xanthones **4a**, **4b**, **4e**, **4f**, and **4h**.

A brief SAR analysis indicates that among the (*E*)-2-styrylchromone-type compounds, only derivatives **3f**, **8** and **10** were capable of inhibit the Aβ aggregation, within a range of 18–66% (Table 2, Figure 6). The replacement of *O*-propargyl for the *N,N*-dipropargyl moiety seems to improve the capacity to inhibit the Aβ aggregation, since it slightly enhanced the antiaggregating activity of compound **8**, when compared to the parent compound **3a** (highlighted in orange, Figure 6). Additionally, the presence of methoxy groups at R^2^ and R^4^ in (*E*)-2-styrylchromone **3f** and the presence of D-ring in **10** increased the antiaggregating activity when compared to (*E*)-2-styrylchromone **3a**, with especial relevance for the last case (highlighted in yellow and green, respectively, Figure 6). 

On the other hand, almost all the tested chromeno[3,4-*b*]xanthones **4a-i** were found to be good Aβ aggregation inhibitors, except for derivative **4a**, probably due to its highly hydrophobic nature. Although the aromatic interactions seem to play a crucial role in Aβ aggregation inhibition, the absence of hydrogen bonding groups may limit the compounds interaction with mature fibrils. Among the chromeno[3,4-*b*]xanthone series, derivative **4b** exhibited the best inhibition against the Aβ aggregation, probably due to its flat aromatic moiety, as well as the presence of methoxy groups, as in both **4e** and **4f** derivatives (highlighted in red, yellow, and blue, respectively, Figure 6). 

Overall, these results seem to be in line with several previous investigations, which stated that small molecules containing single or multiple aromatic rings with hydroxy or methoxy groups, as the chromeno[3,4-*b*]xanthones **4** are capable of inhibit the Aβ aggregation [18,29]. As in the AChE inhibition, the structural rigidification of (*E*)-2-styrylchromones **3** into chromeno[3,4-*b*]xanthones **4** was pivotal for the enhancement of compounds’ antiaggregating activity (Figure 6).

#### 2.2.4. Dual-Target Activity Analysis

The main objective of this work was to design, synthesize and evaluate the biological potential of a small library of chromeno[3,4-*b*]xanthones as first-in-class dual-target agents for AD. The obtained results from the AChE and Aβ aggregation inhibition assays showed that many of the synthesized compounds emerged as single-target agents. Nevertheless, a few other compounds stood out as lead structures for the development of more effective dual-target agents against AD, as shown in Table 3. The obtained results revealed IC_50_ values ranging from 2.9 to 8.7 μM, and inhibitory ranges of 18–70% (at 20 μM) against the AChE and Aβ aggregation, respectively (Table 3). Most compounds were found to have enough binding affinity towards both targets to be considered to be first-in-class leads, being (*E*)-2-styrylchromone **8** the weakest and chromeno[3,4-*b*]xanthone **4b** and (*E*)-2-styrylchromone **10** the strongest compounds.

Conversely, the best results were obtained for compounds **4b** and **10**, which exhibited moderate to good inhibitory activity against AChE and Aβ, with IC_50_ values of 2.9–3.9 μM and inhibitory percentages of 66–70%, respectively (Table 3). The presence of the D-ring in compound **10** seems to be crucial for both anticholinesterase and antiaggregating activity enhancement, as well as the presence of hydrophilic groups (such as methoxy groups) throughout the flat aromatic moiety of compound **4b** (Table 3).

As an overall SAR analysis, the structural rigidification was proven to be of paramount importance also for dual-target activity, since the best dual-target compounds were chromeno[3,4-*b*]xanthones **4**. As discussed above, the structural modification promotes conformational restraint, which may block unwanted conformations, enhancing the compound’s activity towards AChE and Aβ. Additionally, this type of compounds containing single or multiple aromatic rings and hydrophilic groups, such as methoxy groups, have been underlined for their good antiaggregating properties. Finally, the introduction of rigid and hydrophobic groups, such as the extra phenyl ring can promote the interaction with both targets, especially with the hydrophobic region in the active site of AChE. 

## 3. Materials and Methods

### 3.1. Chemistry

#### 3.1.1. General Remarks

Melting points were measured with a Büchi B-540 apparatus. NMR spectra were recorded with a Bruker Avance 300 (300.13 MHz for ^1^H and 75.47 MHz for ^13^C) spectrometer. Chemical shifts (δ) are reported in ppm and coupling constants (*J*) in Hz; the internal standard was TMS. Unequivocal ^13^C assignments were made with the aid of 2D *g*HSQC and *g*HMBC (delays for one-bond-long range *J*_C/H_ couplings were optimized for 145 and 7 Hz, respectively) experiments. Positive-ion ESI mass spectra were acquired with a QTOF 2 instrument [dilution of 1 µL] of the sample in chloroform solution (ca. 10–5 mL) in 200 µL of 0.1% trifluoroacetic acid/methanol solution. Nitrogen was used as the nebuliser gas and argon as the collision gas. The needle voltage was set at 3000 V, with the ion source at 80 °C and the desolvation temperature at 150 °C. The cone voltage was 35 V. Other low- and high-resolution mass spectra (EI, 70 eV) were measured with VG Autospec Q and M spectrometers. Preparative thin-layer chromatography was performed with Merck silica gel (60 DGF254). All chemicals and solvents used were obtained from commercial sources and used as received or dried using standard procedures. MW-assisted reactions were carried out in a CEM Discover SP apparatus.

#### 3.1.2. Synthesis of (*E*)-2-Styrylchromones **3a-i**, **6**, and **10**

To a stirred solution of sodium (110 mg, 5 mmol) in ethanol (5 mL) were added the appropriate 2-methylchromone **1a-d** (1.25 mmol) and the respective aldehydes **2a-c**, **5** or **7** (1.56 mmol). The resulting mixture was stirred at room temperature for 3–6 h (completion of the reaction monitored by TLC). After that period, the reaction mixture was poured into cold water (10 mL) and the pH adjusted to 4 with 10% aqueous HCl. The crude precipitate was collected by filtration, taken in dichloromethane, and purified by silica gel preparative thin-layer chromatography (TLC), using dichloromethane as eluent.

**(*E*)-2-[2-(prop-2-yn-1-yloxy)styryl]-4*H*-chromen-4-one (3a):** yield 340 mg (90%). Both spectroscopic and analytic data are in accordance with those previously published [34].

**(*E*)-7-methoxy-2-[2-(prop-2-yn-1-yloxy)styryl]-4*H*-chromen-4-one (3b):** yield 366 mg (88%). Both spectroscopic and analytic data are in accordance with those previously published [34].

**(*E*)-5-methoxy-2-[2-(prop-2-yn-1-yloxy)styryl]-4*H*-chromen-4-one (3c)**: yield 361 mg (87%). Both spectroscopic and analytic data are in accordance with those previously published [34].

**(*E*)-7,8-dimethoxy-2-[2-(prop-2-yn-1-yloxy)styryl]-4*H*-chromen-4-one (3d):** yield 229 mg (79%); m.p. 154–157 °C. ^1^H NMR (300 MHz, CDCl3): δ = 2.58 (t, 1H, H-3′’, *J* 2.4 Hz), 4.00 (s, 3H, 7-OC*H*3), 4.09 (s, 3H, 8-OC*H*3), 4.79 (d, 2H, H-1′’, *J* 2.4 Hz), 6.26 (s, 1H, H-3), 6.89 (d, 1H, H-α, *J* 16.3 Hz), 6.99- 7.07 (m, 3H, H-6, H-3′, H-5′), 7.35 (ddd, 1H, H-4′, *J* 8.2, 7.4, 1.7 Hz), 7.62 (dd, 1H, H-6′, *J* 7.7 Hz), 7.91 (d, 1H, H-5, *J* 8.9 Hz), 8.02 (d, 1H, H-β, *J* 16.3 Hz) ppm. ^13^C NMR (75 MHz, CDCl3): δ = 56.4 (7-O*C*H3), 56.5 (C-1′’), 61.7 (8- O*C*H3), 76.0 (C-3′’), 78.1 (C-2′’), 109.5 (C-6 or C-3′ or C-5′), 109.9 (C-3), 112.8 (C-6 or C-3′ or C- 5′), 118.9 (C-4a), 121.0 (C-5), 121.1 (C-α), 121.9 (C-6 or C-3′ or C-5′), 124.7 (C-1′), 128.2 (C-6′), 130.9 (C-4′), 131.2 (C-β), 136.8 (C-8), 150.4 (C-7), 156.0 (C-2′), 162.0 (C-2), 178.2 (C-4) ppm. HRMS (ESI^+^): *m/z* [M + H]^+^ calcd for C22H19O5: 363.1233; found 363.1228. (Figures S3 and S4).

**(*E*)-2-[4-methoxy-2-(prop-2-yn-1-yloxy)styryl]-4*H*-chromen-4-one (3e):** yield 378 mg (91%); m.p. 104–107 °C. ^1^H NMR (300 MHz, CDCl3): δ = 2.60 (t, 1H, H-3′’, *J* 2.4 Hz), 3.87 (s, 3H, 5′-OC*H*3), 4.82 (d, 2H, H-1′’, *J* 2.4 Hz), 6.31 (s, 1H, H- 3), 6.59–6.64 (m, 2H), 6.80 (d, 1H, H-α, *J* 16.2 Hz), 7.38 (ddd, 1H, *J* 8.1, 7.0, 1.1 Hz), 7.55 (d, 1H, *J* 8.3 Hz), 7.67 (ddd, 1H, H-7, *J* 8.5, 7.0, 1.7 Hz), 7.85 (d, 1H, H-β, *J* 16.2 Hz), 8.20 (dd, 1H, H-5, *J* 7.9, 1.7 Hz) ppm. ^13^C NMR (125 MHz, CDCl3): δ = 55.3 (5′-O*C*H3), 55.8 (C-1′’), 76.1 (C-3′’), 78.1 (C-2′’), 110.8 (C-3), 117.8 (C-8), 118.7 (C-4a), 123.6 (C-α), 124.8 (C-5), 125.5 (C-6), 127.5 (C-β), 133.3 (C-7), 156.0 (C-8a), 156.2 (C-2′), 159.8 (C-5′), 167.9 (C-2), 178.0 (C-4) ppm. HRMS (ESI^+^): *m/z* [M + H]^+^ calcd for C21H17O4: 333.1121; found 333.1124. (Figures S5 and S6).

**(*E*)-7-methoxy-2-[4-methoxy-2-(prop-2-yn-1-yloxy)styryl]-4*H*-chromen-4-one (3f):** yield 231 mg (51%); m.p. 171–173 °C. ^1^H NMR (300 MHz, CDCl3): δ = 2.60 (t, 1H, H-3′’, *J* 2.4 Hz), 3.87 (s, 3H, 5′-OC*H*3), 3.94 (s, 3H, 7-OC*H*3), 4.83 (d, 2H, H-1′’, *J* 2.4 Hz), 6.24 (s, 1H, H-3), 6.60 (dd, 1H, H-4′, *J* 8.6, 2.3 Hz), 6.64 (d, 1H, H-6′, *J* 2.3 Hz), 6.77 (d, 1H, H-α, *J* 16.2 Hz), 6.93–6.97 (m, 2H, H-6, H-3′), 7.54 (d, 1H, H-8, *J* 8.6 Hz), 7.80 (d, 1H, H-β, *J* 16.2 Hz), 8.10 (d, 1H, H-5, *J* 9.5 Hz) ppm. ^13^C NMR (75 MHz, CDCl3): δ = 55.6 (5′- O*C*H_3_), 55.9 (7-O*C*H_3_), 56.3 (C-1′’), 76.3 (C-3′’), 78.0 (C-2′’), 100.0 (C-6′), 100.4 (C-6 or C-8), 106.5 (C-4′), 109.8 (C-3), 113.9 (C-6 or C-8), 117.8 (C-4a), 118.1 (C-1′), 118.8 (C-α), 127.0 (C- 5), 129.5 (C-3′), 131.3 (C-β), 156.6 (C-8a), 157.2 (C-2′), 157.8 (C-8a), 162.0 (C-5′), 162.5 (C-2), 164.0 (C-7), 178.0 (C-4) ppm. HRMS (ESI^+^): *m/z* [M + H]^+^ calcd for C22H19O5: 363.1233; found 363.1226. (Figures S7 and S8).

**(*E*)-8-methoxy-3-[4-methoxy-2-(prop-2-yn-1-yloxy]styryl)naphthalen-1(4*H*)-one (3g):** yield 222 mg (49%); m.p. 158–161 °C. ^1^H NMR (300 MHz, CDCl3): δ = 2.59 (t, 1H, H-3′’, *J* 2.1 Hz), 3.86 (s, 3H, 5′-OC*H*3), 3.99 (s, 3H, 5-OC*H*3), 4.81 (d, 2H, H-1′’, *J* 2.4 Hz), 6.22 (s, 1H, H-3), 6.58–6.63 (m, 2H, H-3′, H-4′), 6.73 (d, 1H, H-α, *J* 16.2 Hz), 6.79 (d, 1H, H-6′, *J* 8.3 Hz), 7.11 (dd, 1H, H-8, *J* 8.4, 0.9 Hz), 7.51–7.67 (m, 2H, H-6, H-7), 7.76 (d, 1H, H-β, *J* 16.2 Hz) ppm. ^13^C NMR (75 MHz, CDCl3): δ = 55.5 (5′-O*C*H_3_), 56.3 (C-1′’), 56.5 (5- O*C*H_3_), 76.2 (C-3′’), 78.0 (C-2′’), 99.9 (C-4′ or C-6′), 106.1 (C-3′), 106.4 (C-4′ or C-6′), 110.1 (C- 8), 111.3 (C-3), 114.7 (C-4a), 117.8 (C-1′), 118.3 (C-α), 129.5 (C-6 or C-7), 131.3 (C-β), 133.4 (C-6 or C-7), 157.1 (C-2′), 158.1 (C-8a), 159.7 (C-5), 160.6 (C-2), 161.9 (C-4′), 178.5 (C-4) ppm. HRMS (ESI^+^): *m/z* [M + H]^+^ calcd for C22H19O5: 363.1233; found 363.1228. (Figures S9–S10).

**(*E*)-7,8-dimethoxy-2-[4-methoxy-2-(prop-2-yn-1-yloxy)styryl]chroman-4-one (3h):** yield 196 mg (40%); m.p. 185–188 °C. ^1^H NMR (300 MHz, CDCl3): δ = 2.59 (t, 1H, H-3′’, *J* 2.4 Hz), 3.87 (s, 3H, 5′-OC*H*3), 4.00 (s, 3H, 7-OC*H*3), 4.09 (s, 3H, 8-OC*H*3), 4.78 (d, 2H, H-1′’, *J* 2.4 Hz), 6.24 (s, 1H, H-3), 6.58–6.62 (m, 2H), 6.80 (d, 1H, H-α, *J* 16.1 Hz), 7.01 (d, 1H, H- 6, *J* 9.0 Hz), 7.56 (d, 1H, H-5, *J* 9.0 Hz), 7.930–7.96 (m, 2H, H-β, H-4′) ppm. ^13^C NMR (75 MHz, CDCl3): δ = 55.6 (5′-O*C*H3), 56.4 (7-O*C*H3), 56.5 (C-1′’), 61.7 (8-O*C*H3), 76.1 (C-3′’), 109.1 (C-3), 109.6 (C-6), 117.8 (C-1′), 118.5 (C-α), 119.0 (C-4a), 129.4 (C-5), 131.8 (C-β), 157.4 (C-2′), 162.6 (C-2), 178.2 (C-4) ppm. HRMS (ESI^+^): *m/z* [M + H]^+^ calcd for C23H21O6: 393.1333; found 393.1333. (Figures S11 and S12).

**(*E*)-2-[4-bromo-2-(prop-2-yn-1-yloxy)styryl]-4*H*-chromen-4-one (3i):** yield 434 mg (91%); m.p. 171–174 °C. ^1^H NMR (300 MHz, CDCl3): δ = 2.59 (t, 1H, H-3′’, *J* 2.4 Hz), 4.83 (d, 2H, H-1′’, *J* 2.4 Hz), 6.36 (s, 1H, H-3), 6.88 (d, 1H, H-α, *J* 16.2 Hz), 6.98 (d, 1H, H-3′, *J* 8.6 Hz), 7.40 (ddd, 1H, H-6, *J* 8.1, 7.0, 1.0 Hz), 7.45 (dd, 1H, H-8, *J* 8.8, 3.3 Hz), 7.56 (dd, 1H, H-4′, *J* 8.8 Hz), 7.72 (d, 1H, H-6′, *J* 2.4 Hz), 7.83 (d, 1H, H-β, *J* 16.2 Hz), 8.20 (dd, 1H, H-5, *J* 7.9, 1.7 Hz) ppm. ^13^C NMR (75 MHz, CDCl3): δ = 56.5 (C-1′’), 76.7 (C-3′’), 77.2 (C-2′’), 111.0 (C-3), 114.6 (C-6′), 118.0 (C-4′), 114.3 (C-4a), 122.4 (C-α), 125.0 (C-6), 125.7 (C-5), 126.7 (C-1′), 130.4 (C-β), 130.7 (C-3′), 133.1 (C-8), 133.7 (C-7), 154.8 (C-2′), 156.0 (C-8a), 161.6 (C-2), 178.5 (C-4) ppm. HRMS (ESI^+^): *m/z* [M + H]^+^ calcd for C20H14O3Br: 381.0121; found 381.0122. (Figures S13 and S14).

**(*E*)-2-{2-[2-(4-oxo-4*H*-chromen-2-yl)vinyl]phenoxy}acetonitrile (6):** yield 227 mg (60%); m.p. 115–118 °C. ^1^H NMR (300 MHz, CDCl3): δ = 4.76 (d, 2H, H-1′’), 6.37 (s, 1H, H-3), 6.83 (dd, 1H, H-3′, *J* 8.4, 1.0 Hz), 7.06–7.12 (m, 2H, H-α, H-5′), 7.30–7.42 (m, 2H, H-6, H-4′), 7.56 (dd, 1H, H-8, *J* 8.5, 1.1 Hz), 7.61 (dd, 1H, H-6′, *J* 7.7, 1.7 Hz), 7.68 (ddd, 1H, H-7, *J* 8.5, 7.0, 1.6 Hz), 7.96 (d, 1H, H-β, *J* 16.3 Hz), 8.20 (dd, 1H, H-5, *J* 7.9, 1.7 Hz) ppm. ^13^C NMR (75 MHz, CDCl3): δ = 65.8 (C-1′’), 110.6 (C-3), 112.2 (C-3′), 117.9 (C-8), 121.8 (C-α or C-5′), 122.0 (C-α or C-5′), 124.7 (C-4a), 124.8 (C-1′), 124.9 (C-6 or C-4′), 125.0 (C-2′’), 125.6 (C-5), 129.0 (C- 6′), 130.7 (C-6 or C-4′), 132.2 (C-β), 133.5 (C-7), 156.1 (C-2′), 156.4 (C-8a), 162.4 (C-2), 168.4 (C-4) ppm. HRMS (ESI^+^): *m/z* [M + H]^+^ calcd for C19H14O3N: 304.0974; found 304.0970. (Figures S27 and S28).

**(*E*)-2-{2-[di(prop-2-yn-1-yl)amino]styryl}-4*H*-chromen-4-one (8):** yield 223 mg (82%); m.p. 117–119 °C. ^1^H NMR (300 MHz, CDCl3): δ = 2.34 (t, 2H, H-3′’, *J* 2.4 Hz), 4.00 (d, 2H, H-1′’, *J* 2.4 Hz), 6.39 (s, 1H, H-3), 6.83 (d, 1H, H-α, *J* 16.2 Hz), 7.18 (ddd, 1H, H-5′, *J* 8.1, 5.5, 2.7 Hz), 7.35–7.43 (m, 3H, H-6, H-4′, H-3′), 7.56 (dd, 1H, H-8, *J* 8.3, 1.5 Hz), 7.63 (m, 1H, H-6′), 7.68 (ddd, 1H, H-7, *J* 8.7, 7.1, 1.5 Hz), 8.01 (d, 1H, H-β, *J* 16.2 Hz), 8.21 (dd, 1H, H-5, *J* 8.0, 1.7 Hz) ppm. ^13^C NMR (75 MHz, CDCl_3_) = 73.6 (C-3′’), 78.9 (C-2′’), 110.4 (C-3), 118.0 (C-8), 120.9 (C-α), 121.4 (C-5′), 124.2 (C-4a), 124.4 (C-1′), 125.0 (C-6), 125.7 (C-5), 127.7 (C-6), 129.9, (C-6′), 130.1 (C-4′), 133.6 (C-β), 134.1 (C-7), 149.1 (C-2′), 156.1 (C-8a), 162.2 (C-2), 178.5 (C-4) ppm. HRMS (ESI^+^): *m/z* [M + H]^+^ calcd for C23H18O2N: 340.1332; found 340.1332. (Figures S29 and S30).

**(*E*)-2-(2-{[3-(4-methylphenyl)prop-2-yn-1-yl]oxy}styryl)-4*H*-chromen-4-one (10):** yield 383 mg (78%); m.p. 150–153 °C. ^1^H NMR (300 MHz, CDCl3): δ = 2.34 (s, 3H, 4′’’-C*H*3), 5.06 (s, 2H, H-1′’), 6.36 (s, 1H, H-3), 6.91 (d, 1H, H-α, *J* 16.2 Hz), 7.04- 7.12 (m, 3H, H-5′, H-3′’’, H-5′’’), 7.18 (dd, 1H, H-3′, *J* 8.4, 1.1 Hz), 7.33 (d, 2H, H-2′’’, H-6′’’, *J* 8.1 Hz), 7.35–7.41 (m, 2H, H-6, H-4′), 7.55 (dd, 1H, H-8, *J* 8.5, 0.6 Hz), 7.64–7.70 (m, 2H, H-7, H-6′), 8.00 (d, 1H, H-β, *J* 16.2 Hz), 8.20 (dd, 1H, H-5, *J* 8.1, 1.7 Hz) ppm. ^13^C NMR (125 MHz, CDCl_3_) = 21.5 (*C*H_3_), 57.5 (C-1′’), 82.8 (C-3′’), 88.0 (C-2′’), 110.4 (C-3), 113.3 (C-3′), 118.0 (C-8), 119.0 (C-α), 121.0 (C-1′’’), 121.8 (C-5′), 121.8 (C-4′’’), 124.2 (C-4a), 124.8 (C-1′), 124.9 (C-5), 125.6 (C-6), 128.2 (C-6′), 129.1 (C-3′,5′), 131.0 (C-4′), 131.7 (C-2′,6′), 132.3 (C-β), 133.6 (C-7), 139.1 (C-4′’’), 156.1 (C-8a), 156.3 (C-2′), 162.4 (C-2), 178.6 (C-4) ppm. HRMS (ESI^+^): *m/z* [M + H]^+^ calcd for C27H21O3: 393.1485; found 393.1487. (Figures S31 and S32).

#### 3.1.3. Synthesis of Chromeno[3,4-*b*]xanthones (**4a-i**)

The appropriate (*E*)-2-styrylchromone **3a-i** (0.1 mmol) and 1,2,4-trichlorobenzene (1,2,4-TCB) (1 mL) were mixed in a closed glass vessel. The resulting mixture was heated under MW irradiation at 220 °C for 30 min. After that period, chloranil (10 μmol, 2.5 mg) was added to the crude mixture and it was heated under MW irradiation at 80 °C for another 30 min. The resulting crude was diluted in dichloromethane (5 mL) and purified by preparative TLC. A preliminary elution with hexane allows the removal of 1,2,4-TCB from the reaction mixture, which is then followed by a second elution with dichloromethane to isolate the desired product.

**6*H*,8*H*-Chromeno[3,4-*b*]xanthen-8-one (4a):** yield 22 mg (75%). Both spectroscopic and analytic data are in accordance with those previously published [34].

**11-Methoxy-5,6-dihydro-8*H*-naphtho[2,1-*b*]xanthen-8-one (4b):** yield 26 mg (79%). Both spectroscopic and analytic data are in accordance with those previously published [34].

**9-Methoxy-6*H*,8*H*-chromeno[3,4-*b*]xanthen-8-one (4c):** yield 22 mg (67%). Both spectroscopic and analytic data are in accordance with those previously published [34].

**11,12-Dimethoxy-6*H*,8*H*-chromeno[3,4-*b*]xanthen-8-one (4d):** yield 30 mg (83%); m.p. 229–232 °C. ^1^H NMR (300 MHz, CDCl3): δ = 4.03 (s, 3H, 11-OC*H*3), 4.07 (s, 3H, 12-OC*H*3), 5.23 (s, 2H, H-6), 7.02–7.06 (m, 2H, H-1, H-10), 7.13 (td, 1H, H-2, *J* 7.5, 1.2 Hz), 7.35 (ddd, 1H, H-3, J 8.4, 7.5, 1.6 Hz), 7.84 (dd, 1H, H-4, *J* 7.9, 1.6 Hz), 7.87 (s, 1H, H- 14), 8.09–8.11 (m, 2H, H-7, H-9) ppm. ^13^C NMR (75 MHz, CDCl3): δ = 56.5 (11-O*C*H_3_), 61.7 (12- O*C*H3), 68.0 (C-6), 108.7 (C-1 or C-10), 111.10 (C-14), 118.0 (C-1 or C-10), 120.4 (C-7a), 121.5 (C-14a), 122.5 (C-8a), 122.6 (C-7 or C-9), 122.6 (C-2), 124.4 (C-4), 127.7 (C-6a), 131.5 (C-3), 136.4 (C-12), 136.8 (14a), 155.6 (C-4a), 156.5 (13a), 157.7 (C-11), 176.0 (C-8) ppm. HRMS (ESI^+^): *m/z* [M + H]^+^ calcd for C22H17O5: 361.1071; found 361.1072. (Figures S15 and S16). 

**2-Methoxy-6*H*,8*H*-chromeno[3,4-*b*]xanthen-8-one (4e):** yield 17 mg (51%). m.p. 213–216 °C. ^1^H NMR (300 MHz, CDCl3): δ = 3.85 (s, 3H, 2-OC*H*3), 5.21 (s, 2H, H-6), 6.57 (d, 1H, H-1, *J* 2.5 Hz), 6.70 (dd, 1H, H-3, *J* 8.7, 2.5 Hz), 7.38 (ddd, 1H, H-10, *J* 8.0, 7.0, 1.1 Hz), 7.50 (dd, 1H, H-12, *J* 8.5, 1.1 Hz), 7.65 (s, 1H, H-14), 7.70–7.76 (m, 2H, H-4, H-11), 8.09 (s, 1H, H-7), 8.34 (dd, 1H, H-9, *J* 8.0, 1.7 Hz) ppm. ^13^C NMR (75 MHz, CDCl3): δ = 55.5 (2-O*C*H3), 68.2 (C-6), 102.3 (C-4), 109.6 (C-14), 109.8 (C-3), 114.4 (C-7a), 117.9 (C-12), 120.0 (C-6a), 121.9 (C-8a), 122.5 (C-7), 123.9 (C-10), 125.4 (C-1 or C-11), 126.5 (C-9), 131.7 (C-1 or C-11), 150.7 (C-12a), 156.3 (C-12a), 156.7 (C-13a), 157.1 (C-4a), 162.5 (C-2), 176.6 (C-8) ppm. HRMS (ESI^+^): *m/z* [M + H]^+^ calcd for C21H15O4: 331.0970; found 331.0980. (Figures S17 and S18).

**2,11-Dimethoxy-6*H*,8*H*-chromeno[3,4-*b*]xanthen-8-one (4f):** yield 22 mg (61%). m.p. 225–228 °C. ^1^H NMR (300 MHz, CDCl3): δ = 3.85 (s, 3H, 2-OC*H*3), 3.95 (s, 3H, 11-OC*H*3), 5.21 (s, 2H, H-6), 6.58 (d, 1H, H-1, *J* 2.5 Hz), 6.70 (dd, 1H, H-10, *J* 8.8, 2.5 Hz), 6.90 (d, 1H, H-12, *J* 2.5 Hz), 6.96 (dd, 1H, H-3, *J* 8.8, 2.5 Hz), 7.62 (s, 1H, H-14), 7.71 (d, 1H, H-4, *J* 8.8 Hz), 8.08 (s, 1H, H-7), 8.25 (d, 1H, H-9, *J* 8.8 Hz) ppm. ^13^C NMR (75 MHz, CDCl_3_) = 55.5 (2-O*C*H_3_), 55.9 (11-O*C*H_3_), 68.3 (C-6), 100.3 (C-12), 102.3 (C-4), 109.4 (C-14), 109.8 (C-3), 113.2 (C-1), 114.4 (C-7a), 115.8 (C-8a), 118.9 (C-7a), 120.1 (C-6a), 122.5 (C-14b), 125.4 (C-7), 126.5, 128.3, 136.8, 143.2, 156.7 (C-12a), 157.0 (C-4a), 158.1 (C-11), 162.5 (C-11), 165.0 (C-2), 175.7 (C-8) ppm. HRMS (ESI^+^): *m/z* [M + H]^+^ calcd for C22H17O5: 361.1071; found 361.1072. (Figures S19 and S20).

**2,9-Dimethoxy-6*H*,8*H*-chromeno[3,4-*b*]xanthen-8-one (4g):** yield 19 mg (53%). m.p. 224–227 °C. ^1^H NMR (300 MHz, CDCl3): δ = 3.85 (s, 3H, 2-OC*H*3), 4.03 (s, 3H, 9-OC*H*3), 5.20 (s, 2H, H-6), 6.58 (d, 1H, H-1, *J* 2.6 Hz), 6.69 (dd, 1H, H-3, *J* 8.7, 2.6 Hz), 6.81 (dd, 1H, H-10, *J* 8.4, 1.0 Hz), 7.07 (dd, 1H, H-12, *J* 8.4, 1.0 Hz), 7.58 (s, 1H, H-14), 7.61 (t, 1H, H-11, *J* 8.4 Hz), 7.70 (d, 1H, H-4, *J* 8.7 Hz), 8.05 (s, 1H, H-7) ppm. ^13^C NMR (75 MHz, CDCl3): δ = 55.5 (2-O*C*H3), 56.5 (9-O*C*H3), 68.3 (C-6), 102.3 (C-1), 105.5 (C-10), 109.1 (C-14), 109.8 (C-3), 111.0 (-12), 112.6 (C-8a), 121.2 (C-7a), 122.7 (C-7), 125.4 (C-4), 126.5 (C-6a), 134.8 (C-11), 136.7 (C-14a), 155.5 (C-12a), 157.0 (C-4a), 158.2 (C-13a), 160.8 (C-9), 162.4 (C-2), 176.0 (C-8) ppm. HRMS (ESI^+^): *m/z* [M + H]^+^ calcd for C22H17O5: 361.1068; found 361.1069. (Figures S21 and S22).

**2,11,12-trimethoxy-6*H*,8*H*-chromeno[3,4-*b*]xanthen-8-one (4h):** yield 18 mg (45%). m.p. 231–234 °C. ^1^H NMR (300 MHz, CDCl3): δ = 3.85 (s, 3H, 2-OC*H*3), 4.03 (s, 3H, 12-OC*H*3), 4.06 (s, 3H, 11-OC*H*3), 5.21 (s, 2H, H-6), 6.57 (d, 1H, H-1, *J* 2.5 Hz), 6.70 (dd, 1H, H-3, *J* 8.9, 2.5 Hz), 7.02 (d, 1H, H-4, *J* 8.9Hz), 7.72–7.75 (m, 2H, H-10, H-14), 8.07–8.10 (m, H-7, H-9) ppm. ^13^C NMR (75 MHz, CDCl3): δ = 55.5 (2-O*C*H_3_), 56.4 (12-O*C*H_3_), 61.7 (11-O*C*H_3_), 68.2 (C-6), 102.3 (C-1), 108.6 (C-4), 109.8 (C-3), 109.9 (C-10 or C-14), 119.6 (C-7a), 122.4 (C-7 or C-9), 122.5 (C-8a), 125.5 (C-10 or C-14), 126.6 (C-6a), 136.4 (C-11), 137.1 (C-14a), 150.1 (C-12a), 156.6 (C-4a), 157.1 (C-13a), 157.6 (C-12), 162.5 (C-2),176.0 (C- 8) ppm. HRMS (ESI^+^): *m/z* [M + H]^+^ calcd for C23H19O6: 391.1182; found 391.1185. (Figures S23 and S24).

**2-bromo-6*H*,8*H*-chromeno[3,4-*b*]xanthen-8-one (4i):** yield 20 mg (52%). m.p. 211–215 °C. ^1^H NMR (300 MHz, CDCl3): δ = 5.21 (s, 2H, H-6), 6.93 (d, 1H, H-4, *J* 8.7 Hz), 7.37–7.43 (m, 2H, H-3, H-10), 7.50 (dd, 1H, H-12, *J* 8.5, 1.0 Hz), 7.71 (s, 1H, H-14), 7.74 (ddd, 1H, H-11, *J* 8.5, 7.0, 1.7 Hz), 7.89 (d, 1H, H-1, *J* 2.3 Hz), 8.11 (s, 1H, H-7), 8.32 (dd, 1H, H-9, *J* 7.9, 1.7 Hz) ppm. ^13^C NMR (75 MHz, CDCl3): δ = 68.0 (C-6), 111.2 (C-14), 118.0 (C-12), 119.8 (C-4), 121.4 (C-7a), 121.8 (C-8a), 122.9 (C-7), 124.2 (C-3 or C-10), 126.7 (C-9), 127.1 (C- 1), 127.2 (C-6a), 134.0 (C-3 or C-10), 135.0 (C-11), 135.6 (C-14a), 154.6 (C-4a), 156.2 (C-12a), 156.4 (C-13a), 176.5 (C-8) ppm. HRMS (ESI^+^): *m/z* [M + H]^+^ calcd for C20H12O3Br: 378.9964; found 378.9966. (Figures S25 and S26).

### 3.2. AChE Inhibition

The inhibitory activity of the synthesized compounds towards AChE was evaluated spectrophotometrically by a 96-well microplate modified Ellman’s method. The enzyme (AChE) from *Electrophorus electricus* (*ee*AChE) stock solution was prepared as 7.4 U/mL in phosphate buffer, at pH 7. The stock solutions for the target compounds (0.1 M) were prepared in DMSO. The assay solution consisted of 0.0005 M 5,5′-dithiobis(2-nitrobenzoic acid) (DTNB), 0.013 U/mL *ee*AChE, and 0.0025 M acetylthiocholine iodide ATC (substrate for AChE) in 0.1 M phosphate buffer, at pH 7. The reaction mixture was prepared with 100 μL of the enzyme (*ee*AChE) and 50 μL of the test compound (or DMSO/water, not exceeding 0.2% of DMSO in the wells; i.e., blank samples). After 5 min of the pre-incubation period at 37 °C, 50 μL of acetylthiocholine iodide ATC and 50 μL of 5,5′-dithiobis(2-nitrobenzoic acid) (DTNB) were added to each well, thus initiating the enzymatic reaction. The absorbance values were measured at 415 nm, for a total of 7.5 min, at every 2.5 min, using a microplate reader (Synergy multi-mode reader; BioTek). All compounds were tested at a screening concentration of 20 μM. The enzyme inhibition was calculated using the 100 (S/B) × 100 formula, where S and B are the enzyme activities with and without the test compound, respectively. The IC_50_ values were determined for the compounds with an inhibitory activity higher than 50% at 20 μM, using seven different concentrations of 12.5, 10, 7.5, 5, 2.5, 1.0 and 0.5 μM. The absorbance was measured at seven different concentrations of inhibitor, converted to % inhibition of the enzyme and plotted against the applied inhibitor concentration, using a nonlinear regression. Donepezil was used as the reference compound. All the reactions were performed in triplicate. 

### 3.3. Molecular Docking

Molecular docking calculations, using the GOLD software were carried out to determine the molecular interactions between the compounds and acetylcholinesterase [31]. The pdb codes 1C2O and 4BDT were used as structure inputs for the *ee*AChE and hAChE, respectively [35,36]. The missing atoms and hydrogen atoms were added using the tleap software from the Amber18 package and the protonation states carefully determined using Propka3.1 program [37,38]. The results indicated that all residues have a standard pKa and all the histidines were protonated at epsilon nitrogen. The compounds were designed using the Marvin Suite software. To ensure enough space for the docking search we defined a cavity with a radius of 15 Å centered on the position occupied by the huprine ligand. For each compound, 20 runs were requested using the ChemScore scoring function, allowing full flexibility of the ligand and the protein active site.

### 3.4. Aβ Aggregation Inhibition

The inhibition of Aβ1-42 aggregation was determined through a ThT-based fluorimetric assay. Initially, the Aβ-peptides (1 mg) were pre-treated with 1,1,1,3,3,3,-hexafluoro-2-propanol (HFIP), kept at room temperature overnight, aliquoted, and dried under vacuum. The Aβ-peptides stock solutions (111 μM) were then prepared in ammonium hydroxide 0.1% (NH_4_OH) prior to the fluorescence assay. The stock solutions of all tested compounds (400 μM) were prepared in DMSO, while the stock solution of ThT (400 μM) was prepared in distilled water (1 mL). The Aβ aggregation was monitored using a standard 96-well plates, in the Synergy multi-mode reader (BioTek) and setting the excitation and emission wavelengths at 440 and 485 nm, respectively. Samples were prepared by diluting the stock solutions in PBS pH 7.4 to a final Aβ peptides concentration of 10 μM, 20 μM of ThT and 20 μM of the test compound (maximum final DMSO content: 5% *v*/*v*), with a final volume of 100 μL. The assays were performed at room temperature. The fluorescence data was recorded over 24 h with 5 s shaking (360 cpm) prior to each reading and then plotted against the time, using a nonlinear regression. Phenol red was used as the reference compound. Each condition was performed in triplicate, and values were averaged. The estimation of the inhibitory potency (%) was carried out by comparing fluorescence values at the plateau. 

## 4. Conclusions

In this investigation, we have focused our efforts on the design, synthesis, and biological evaluation of a family of chromeno[3,4-*b*]xanthones and their respective precursors, (*E*)-2-styrylchromones, as first-in-class dual-target inhibitors of AChE and Aβ aggregation. We found that almost half of the 21 tested compounds exhibited inhibitory activity against the selected biological targets in the micromolar range, and some of them even showed dual activity towards AChE and Aβ aggregation. Their binding modes towards AChE were determined through molecular docking and the results showed that most of the compounds bound to the enzyme through hydrogen bonds and π-stacking interactions between the main scaffold and Tyr341 and Trp286 aromatic sidechains present in the binding pocket. The best results were obtained for compounds **3d** and **4a**, which effectively bound to AChE, mostly through the formation of a perfect stacking with its Tyr341 side chain in the first case and through the extensive stacking with three aromatic side chains, Phe295, Tyr337 and Trp86, in the second case. The obtained results for each biological test individually showed that chromeno[3,4-*b*]xanthones **4a**, **4b**, **4e**, **4f**, (*E*)-2′-propargyloxy-2-styrylchromone **3d** and (*E*)-2-styrylchromone **10** exhibited good inhibition against AChE with IC_50_ values 2.1–6.9 μM, while chromeno[3,4-*b*]xanthones **4b**, **4e**, **4f** and (*E*)-2-styrylchromone **10** were moderate inhibitors of Aβ aggregation, within a percentage range of 52–70%, at 20 μM. Further investigation to establish the mode of action of such compounds is ongoing in our laboratory, as well as a complete SAR profile with a larger number of analogues. Nonetheless, we have identified a new family of compounds as a template that needs to be developed to find lead molecules. Thereafter, the identification of a lead compound that is currently underwent in our lab, will be furthered by a critical evaluation of its blood-brain barrier (BBB) penetrance and its toxicological effects using specific approaches. Among the tested compounds, chromeno[3,4-*b*]xanthone **4b** and (*E*)-2-styrylchromone **10** should be outlined as the most promising ones, due to their well-balanced activity towards both selected targets. These two derivatives will be used as templates for the development of more potent multitarget compounds for AD-related targets. 

## Figures and Tables

**Table 1:** Inhibition of *ee*AChE by (*E*)-2-styrylchromones **3a-i**, **6**, **8** and **10**, and chromeno[3,4-*b*]xanthones **4a-i**.

Compound	Structure	R^1^	R^2^	R^3^	R^4^	*ee*AChE [IC_50_ (μM)] ^[a]^
**3a**	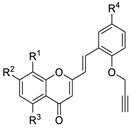	H	H	H	H	**9.5 ± 0.3**
**3b**		H	OMe	H	H	>20
**3c**		H	H	OMe	H	>20
**3d**		OMe	OMe	H	H	**2.4 ± 0.1**
**3e**		H	H	H	OMe	>20
**3f**		H	OMe	H	OMe	>20
**3g**		H	H	OMe	OMe	>20
**3h**		OMe	OMe	H	OMe	>20
**3i**		H	H	H	Br	>20
**6**	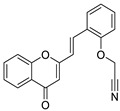	-	-	-	-	>20
**8**	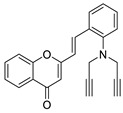	-	-	-	-	**8.7 ± 0.2**
**10**	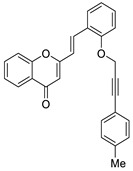	-	-	-	-	**2.9 ± 0.1**
**4a**	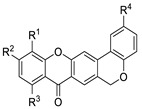	H	H	H	H	**2.1 ± 0.2**
**4b**		H	OMe	H	H	**3.9 ± 0.1**
**4c**		H	H	OMe	H	>20
**4d**		OMe	OMe	H	H	>20
**4e**		H	H	H	OMe	**6.9 ± 0.3**
**4f**		H	OMe	H	OMe	**4.8 ± 0.1**
**4g**		H	H	OMe	OMe	>20
**4h**		OMe	OMe	H	OMe	>20
**4i**		H	H	H	Br	>20
**Donepezil**						0.0156 ± 0.0016

^[a]^ Values are expressed as the means ± the standard error of the mean (SEM) of at least three experiments (*n* = 3), each performed in triplicate.

**Table 2:** Inhibitory activity towards Aβ aggregation of (*E*)-2-styrylchromones **3a**, **3b**, **3d**, **3f**, **6**, **8** and **10**, and chromeno[3,4-*b*]xanthones **4a**, **4b**, **4e**, **4f**, and **4h**.

Compound	Structure	R^1^	R^2^	R^3^	R^4^	Aβ_1-42_ Aggregation (% Inhib. at 20 μM)
**3a**	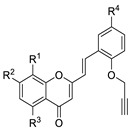	H	H	H	H	<10 ^[a]^
**3b**		H	OMe	H	H	<10 ^[a]^
**3d**		OMe	OMe	H	H	<10 ^[a]^
**3f**		H	OMe	H	OMe	48
**8**	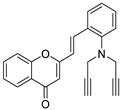	-	-	-	-	18
**10**	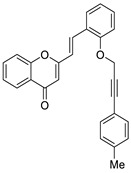	H	H	H	H	66
**4a**	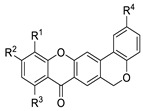	H	H	H	H	<10 ^[a]^
**4b**		H	OMe	H	H	70
**4e**		H	H	H	OMe	61
**4f**		H	OMe	H	OMe	52
**Phenol Red**						20

^[a]^ No inhibition observed.

**Table 3:** Dual-target activity towards *ee*AChE and Aβ aggregation of (*E*)-2-styrylchromones **8** and **10**, and chromeno[3,4-*b*] xanthones **4b**, **4e**, and **4f**.

Compound	Structure	R^1^	R^2^	R^3^	R^4^	*ee*AChE (IC_50_ [μM]) ^[a]^	Aβ_1-42_ Aggregation (% Inhib. at 20 μM)
**8**	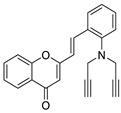	-	-	-	-	8.7 ± 0.2	18
**10**	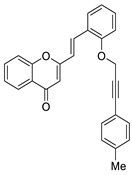	-	-	-	-	2.9 ± 0.1	66
**4b**	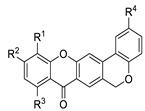	H	OMe	H	H	3.9 ± 0.1	70
**4e**		H	H	H	OMe	6.9 ± 0.3	61
**4f**		H	OMe	H	OMe	4.8 ± 0.1	52

^[a]^ Values are expressed as the means ± the standard error of the mean (SEM) of at least three experiments (*n* = 3), each performed in triplicate.

## Data Availability

Not applicable.

## Supplementary Materials

The following are available online at https://www.mdpi.com/article/10.3390/ijms22084145/s1. Schemes S1–S4: Synthesis of intermediary compounds **1a-d**, **2a-c**, **5**, **7**, and **9**. Figure S1: docking model validation. Figures S2–S32: ^1^H and ^13^C NMR spectra of all synthesized new compounds.
