# Supplementary material for: Chromeno[3,4-b]xanthones as First-in-Class AChE and Aβ Aggregation Dual-Inhibitors
Source: Int J Mol Sci. 2021 Apr 16;22(8):4145. doi: 10.3390/ijms22084145 (PMC8072597; doi:10.3390/ijms22084145)
Supplement: Supplementary file 1 [file ijms-22-04145-s001.zip › ijms-1149668-supplementary.pdf]

Chromeno[3,4-*b*]xanthones as first-in-class AChE and Aβ aggregation dual-inhibitors

Daniela Malafaia [1], Ana Oliveira [2], Pedro A. Fernandes [2], Maria J. Ramos [2], Hélio M. T. Albuquerque [1,*] and Artur M. S. Silva [1,*]

[1] LAQV-REQUIMTE, Department of Chemistry, University of Aveiro, Campus de Santiago, 3810-193, Aveiro, Portugal; danielamalafaia@ua.pt (D.M.)

[2] LAQV-REQUIMTE, Computational Biochemistry Laboratory, Department of Chemistry and Biochemistry, Faculty of Sciences, University of Porto, Rua do Campo Alegre, s/n, 4169-007 Porto, Portugal; anoliveira@fc.up.pt (A.O.); pafernan@fc.up.pt (P.A.F.); mjramos@fc.up.pt (M.J.R)

* Correspondence: helio.albuquerque@ua.com (H.M.T. Albuquerque); artur.silva@ua.pt (A.M.S. Silva)

Contents:

Scheme S1. Synthesis of 2-methylchromones **1a-d**. 2
Scheme S2. Synthesis of aldehydes **2a-c** and **5**. 3
Scheme S3. Synthesis of aldehyde **7**. 3
Scheme S4. Synthesis of aldehyde **9**. 3

Figure S1. Validation of the docking protocol: (a) Sequence alignment between the eeAChE (PDB code 1C2O) and the hAChE (PDB code 4BDT) and (b) Docking pose of huprine. The figure shows a perfect superposition between the co-crystalized ligand (yellow) and the pose found by the gold software (blue) in the hAChE structure. 4
Figure S2: Superimposition of the acetylcholinesterase human and the *E. electricus* binding sites. The x-ray human structure, in green, is complexed with the co-crystalized huprine W ligand (in yellow), and the *E. electricus* x-ray is shown in gray. In sticks, we represent all the residues within a radius of 5 Å from the huprine W ligand. 4
Figure S3. ^{1}H NMR spectrum of (E)-2-styrylchromone (**3d**) (300 Mhz, $CDCl_3$). 5
Figure S4. ^{13}C NMR spectrum of (E)-2-styrylchromone (**3d**) (75 Mhz, $CDCl_3$). 5
Figure S5. ^{1}H NMR spectrum of (E)-2-styrylchromone (**3e**) (300 Mhz, $CDCl_3$). 6
Figure S6. ^{13}C NMR spectrum of (E)-2-styrylchromone (**3e**) (125 Mhz, $CDCl_3$). 6
Figure S7. ^{1}H NMR spectrum of (E)-2-styrylchromone (**3f**) (300 Mhz, $CDCl_3$). 7
Figure S8. ^{13}C NMR spectrum of (E)-2-styrylchromone (**3f**) (75 Mhz, $CDCl_3$). 7
Figure S9. ^{1}H NMR spectrum of (E)-2-styrylchromone (**3g**) (300 Mhz, $CDCl_3$). 8
Figure S10. ^{13}C NMR spectrum of (E)-2-styrylchromone (**3g**) (75 Mhz, $CDCl_3$). 8
Figure S11. ^{1}H NMR spectrum of (E)-2-styrylchromone (**3g**) (300 Mhz, $CDCl_3$). 9
Figure S12. ^{13}C NMR spectrum of (E)-2-styrylchromone (**3h**) (125 Mhz, $CDCl_3$). 9
Figure S13. ^{1}H NMR spectrum of (E)-2-styrylchromone (**3i**) (300 Mhz, $CDCl_3$). 10
Figure S14. ^{13}C NMR spectrum of (E)-2-styrylchromone (**3i**) (75 Mhz, $CDCl_3$). 10
Figure S15. ^{1}H NMR spectrum of chromeno[3,4-b]xanthone (**4d**) (300 Mhz, $CDCl_3$). 11
Figure S16. ^{13}C NMR spectrum of chromeno[3,4-b]xanthone (**4d**) (75 Mhz, $CDCl_3$). 11
Figure S17. ^{1}H NMR spectrum of chromeno[3,4-b]xanthone (**4e**) (300 Mhz, $CDCl_3$). 12
Figure S18. ^{13}C NMR spectrum of chromeno[3,4-b]xanthone (**4e**) (75 Mhz, $CDCl_3$). 12
Figure S19. ^{1}H NMR spectrum of chromeno[3,4-b]xanthone (**4f**) (300 Mhz, $CDCl_3$). 13
Figure S20. ^{13}C NMR spectrum of chromeno[3,4-b]xanthone (**4f**) (75 Mhz, $CDCl_3$). 13
Figure S21. ^{1}H NMR spectrum of chromeno[3,4-b]xanthone (**4g**) (300 Mhz, $CDCl_3$). 14
Figure S22. ^{13}C NMR spectrum of chromeno[3,4-b]xanthone (**4g**) (75 Mhz, $CDCl_3$). 14

Figure S23. ^{1}H NMR spectrum of chromeno[3,4-b]xanthone (**4h**) (300 Mhz, $CDCl_3$). 15
Figure S24. ^{13}C NMR spectrum of chromeno[3,4-b]xanthone (**4h**) (75 Mhz, $CDCl_3$). 15
Figure S25. ^{1}H NMR spectrum of chromeno[3,4-b]xanthone (**4i**) (300 Mhz, $CDCl_3$). 16
Figure S26. ^{13}C NMR spectrum of chromeno[3,4-b]xanthone (**4i**) (75 Mhz, $CDCl_3$). 16
Figure S27. ^{1}H NMR spectrum of (E)-2-styrylchromone (**6**) (300 Mhz, $CDCl_3$)........................... 17
Figure S28. ^{13}C NMR spectrum of (E)-2-styrylchromone (**6**) (75 Mhz, $CDCl_3$)............................ 17
Figure S29. ^{1}H NMR spectrum of (E)-2-styrylchromone (**8**) (300 Mhz, $CDCl_3$)........................... 18
Figure S30. ^{13}C NMR spectrum of (E)-2-styrylchromone (**8**) (75 Mhz, $CDCl_3$)............................ 18
Figure S31. ^{1}H NMR spectrum of (E)-2-styrylchromone (**10**) (500 Mhz, $CDCl_3$)......................... 19
Figure S32. ^{13}C NMR spectrum of (E)-2-styrylchromone (**10**) (125 Mhz, $CDCl_3$)........................ 19

General procedure for the synthesis of 2-methylchromones 1a-d.

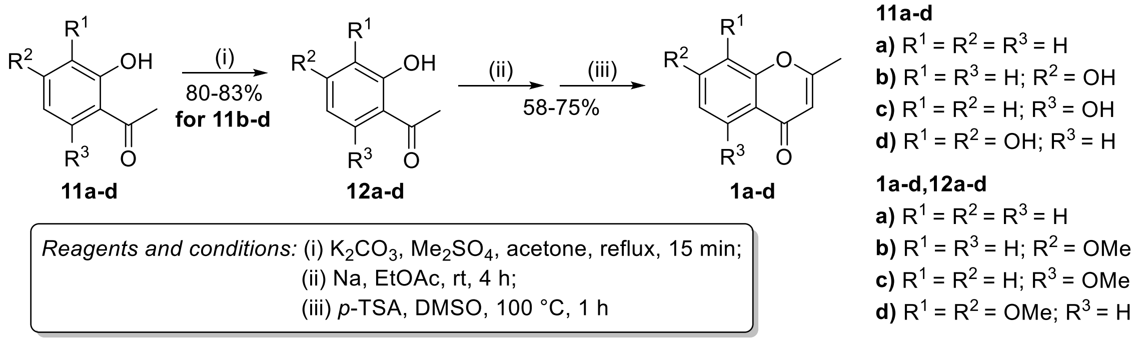

Scheme S1. Synthesis of 2-methylchromones **1a-d**.

To a solution of the appropriate 2'-hydroxyacetophenone **12a-d** (30 mmol) in ethyl acetate (60 mL) was added sodium (4.05 g, 176 mmol). The mixture was stirred at room temperature for 4 h. After that period, the mixture was poured into cold water and the pH adjusted to 4 with HCl (10%). The organic phase was then extracted with CH_2Cl_2 (3 x 100 mL), dried with anhydrous sodium sulfate and the solvent evaporated under reduced pressure, to obtain the β-diketone crude. The resulting β-diketone crude was subsequently dissolved in DMSO (10 mL) and *p*-TSA (0.7 g, 4 mmol) was added. The resulting mixture was stirred at 100 °C for 1 h. After that period, the mixture was poured into cold water and the organic phase was extracted with ethyl acetate (3 x 50 mL), dried with anhydrous sodium sulfate and the solvent evaporated under reduced pressure. The residue was then purified by silica-gel column chromatography, using dichloromethane as eluent.[1]

General procedure for the synthesis of aldehydes 2a-c and 5.

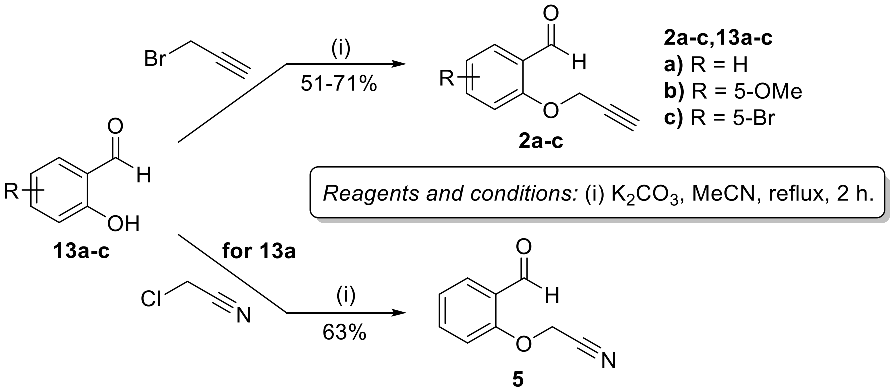

Scheme S2. Synthesis of aldehydes **2a-c** and **5**.

To a solution of the appropriate salicylaldehyde **13a-c** (33 mmol) in acetonitrile (100 mL) was added potassium carbonate (6.84 g, 49.5 mmol) and propargyl bromide or chloroacetonitrile (36.3 mmol). The resulting mixture was refluxed for 2 h. After that period, the mixture was poured into cold water and the pH adjusted to 4 with dilute HCl (10%). The precipitate was recovered in a pure form by filtration and washed with water (50 mL).

General procedure for the synthesis of aldehyde 7.

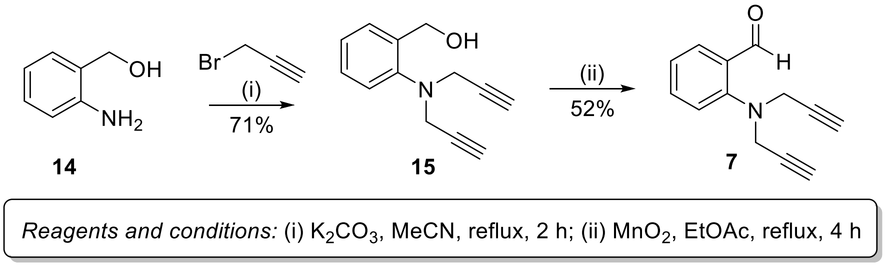

Scheme S3. Synthesis of aldehyde **7**.

To a solution of (2-aminophenyl)methanol **14** (40 mmol) in acetonitrile (100 mL) was added potassium carbonate (11.89 g, 100 mmol) and propargyl bromide (8.6 mL, 100 mmol). The resulting mixture was refluxed for 2 h. After that period, the mixture was poured into cold water and the pH adjusted to 4 with HCl (10%). The precipitate was recovered in a pure form by filtration and washed with water (50 mL). The resulting alcohol **15** (4,00 g, 20 mmol) was dissolved in ethyl acetate (30 mL) and MnO_2 (8.70 g, 100 mmol) was added and the mixture refluxed for 4 h. The resulting slurry was passed through Celite to remove the excess MnO_2, and then purified by silica-gel column chromatography, using dichloromethane as eluent.[2]

General procedure for the synthesis of aldehyde 9.

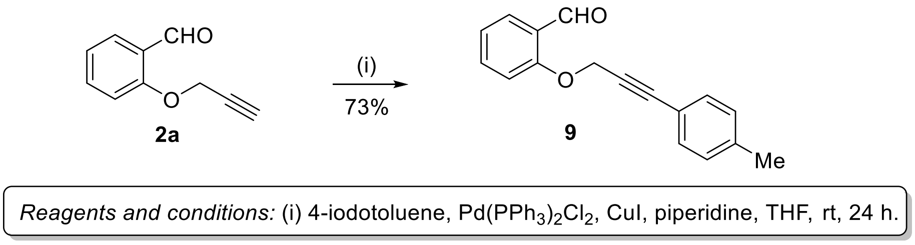

Scheme S4. Synthesis of aldehyde **9**.

To a solution of *O*-propargylated aldehyde **2a** (400 mg, 2 mmol), 4-iodotoluene (2.2 equiv) and piperidine (4.0 equiv) in dry THF (8 mL) under nitrogen atmosphere were added $Pd(PPh_3)_2Cl_2$ (0.06 equiv) and CuI (0.12 equiv). The resulting mixture was stirred at room temperature for 24 h. After that period, the solvent was evaporated under reduced pressure and the crude residue purified by preparative thin layer chromatography (TLC), using a mixture of hexane and dichloromethane as eluent (Hex/CH_2Cl_2, 2:1).

Docking model validation

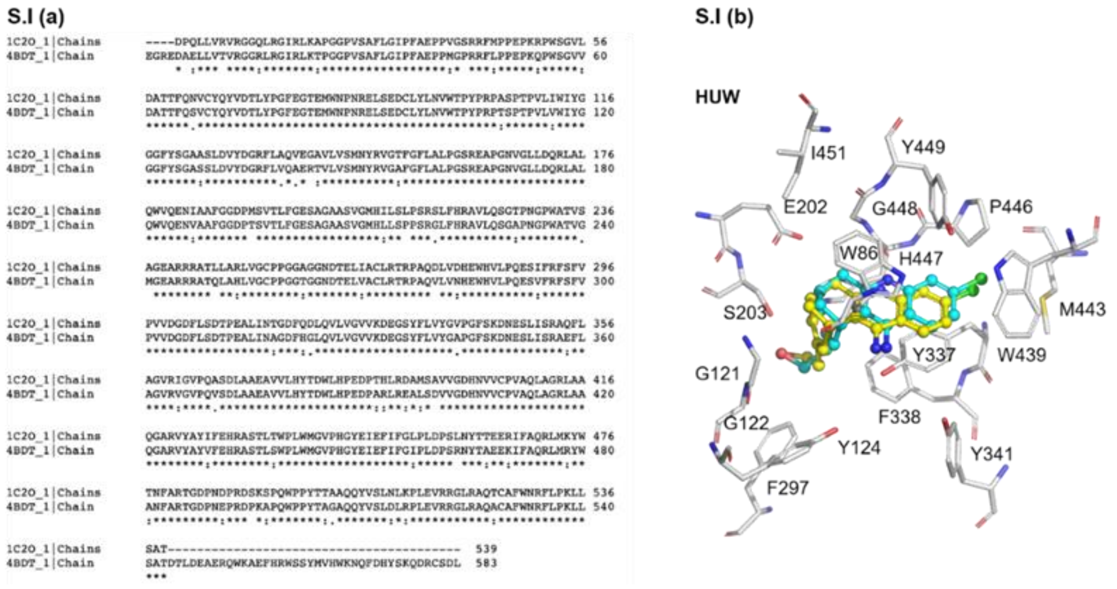

Figure S1. Validation of the docking protocol: (a) Sequence alignment between the eeAChE (PDB code 1C2O) and the hAChE (PDB code 4BDT) and (b) Docking pose of huprine. The figure shows a perfect superposition between the co-crystalized ligand (yellow) and the pose found by the gold software (blue) in the hAChE structure.

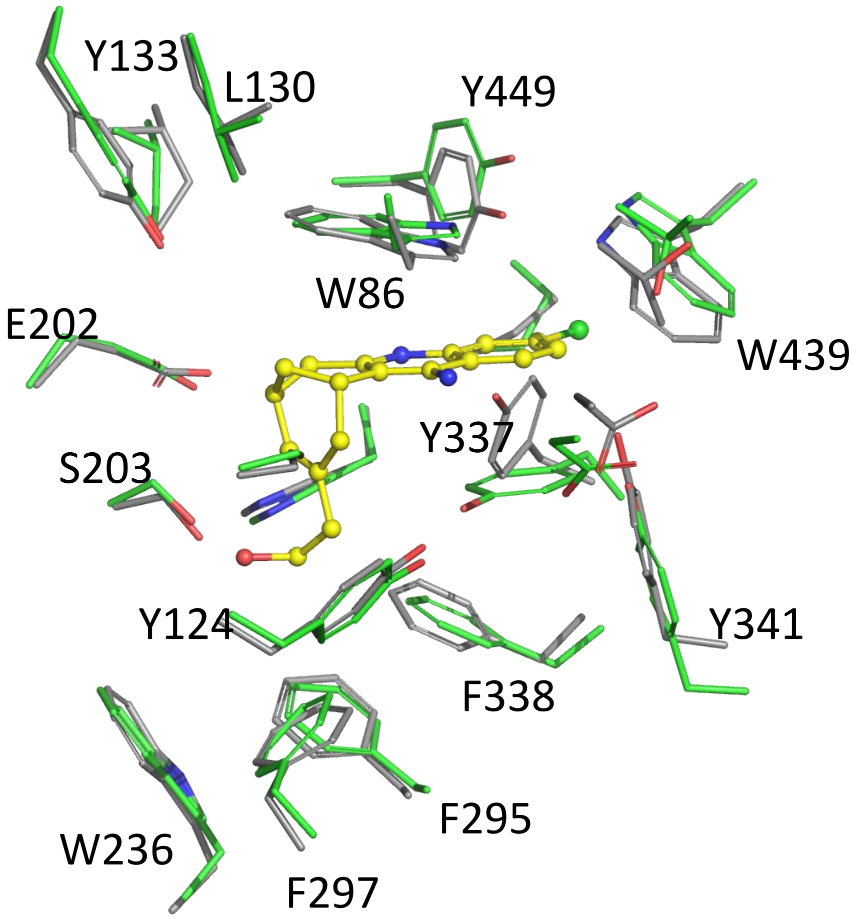

Figure S2: Superimposition of the acetylcholinesterase human and the *E. electricus* binding sites. The x-ray human structure, in green, is complexed with the co-crystalized huprine W ligand (in yellow), and the *E. electricus* x-ray is shown in gray. In sticks, we represent all the residues within a radius of 5 Å from the huprine W ligand.

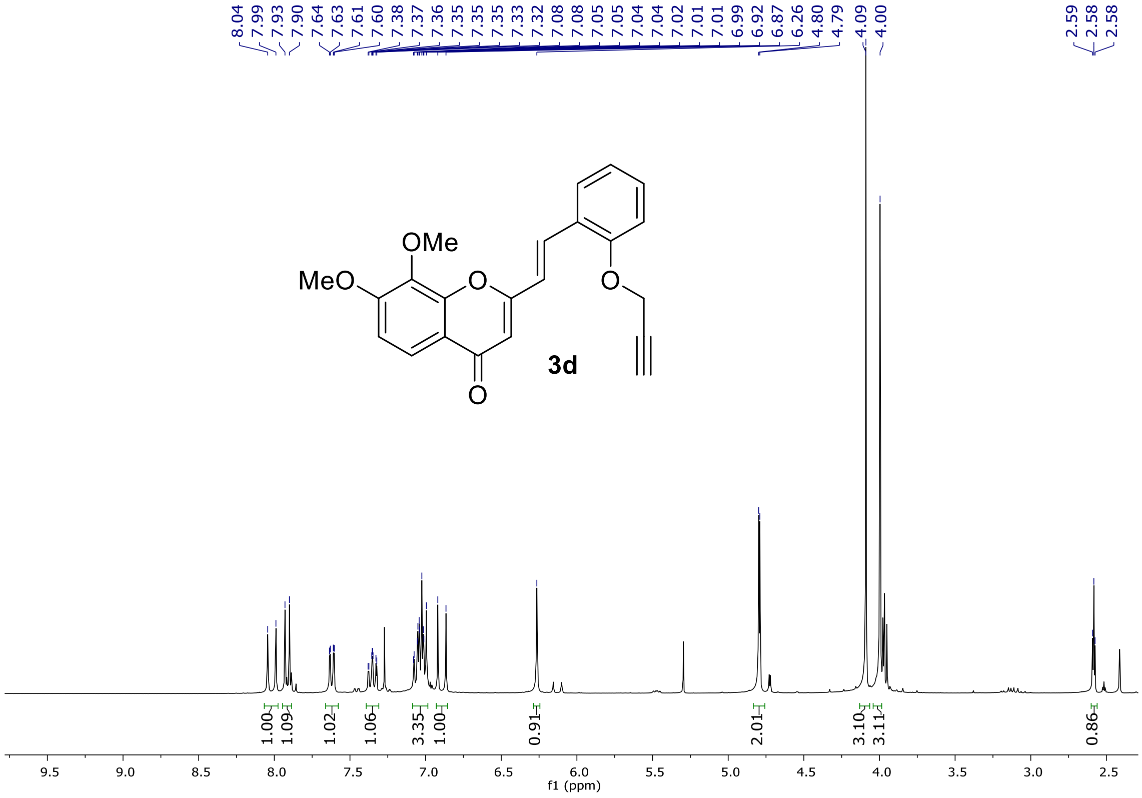

Figure S3. ^{1}H NMR spectrum of (*E*)-2-styrylchromone (**3d**) (300 Mhz, $CDCl_3$).

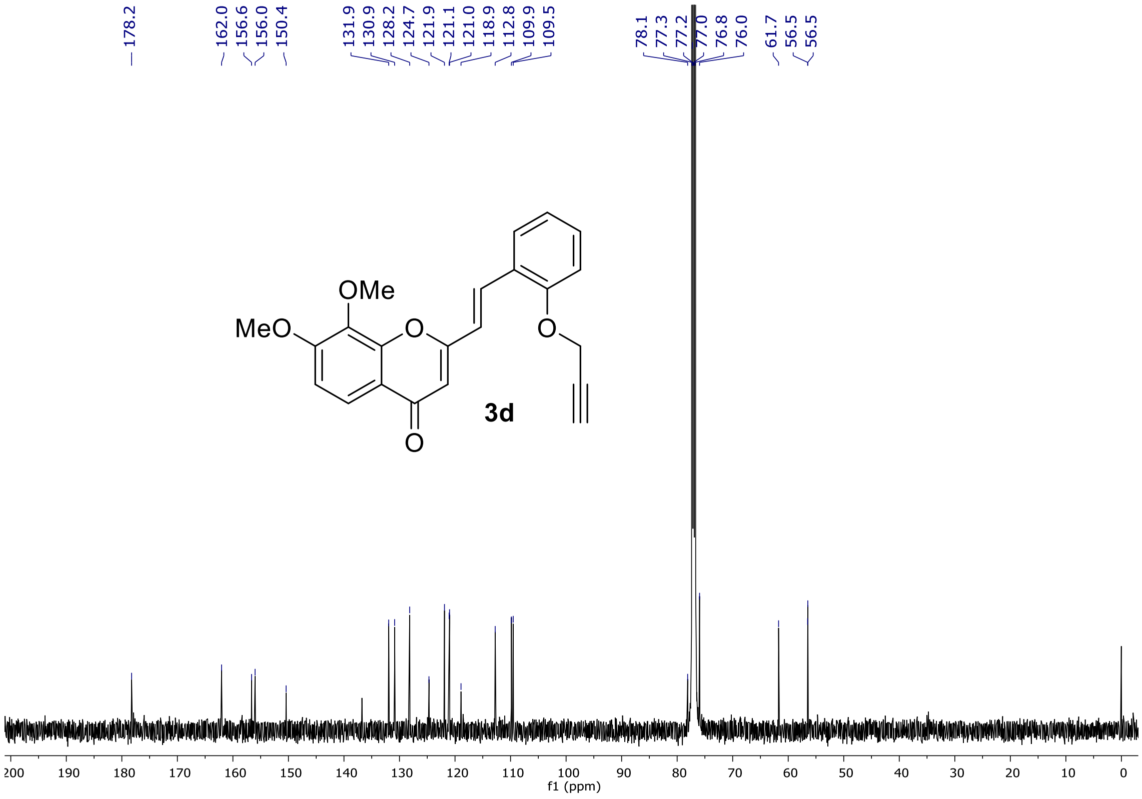

Figure S4. ^{13}C NMR spectrum of (*E*)-2-styrylchromone (**3d**) (75 Mhz, $CDCl_3$).

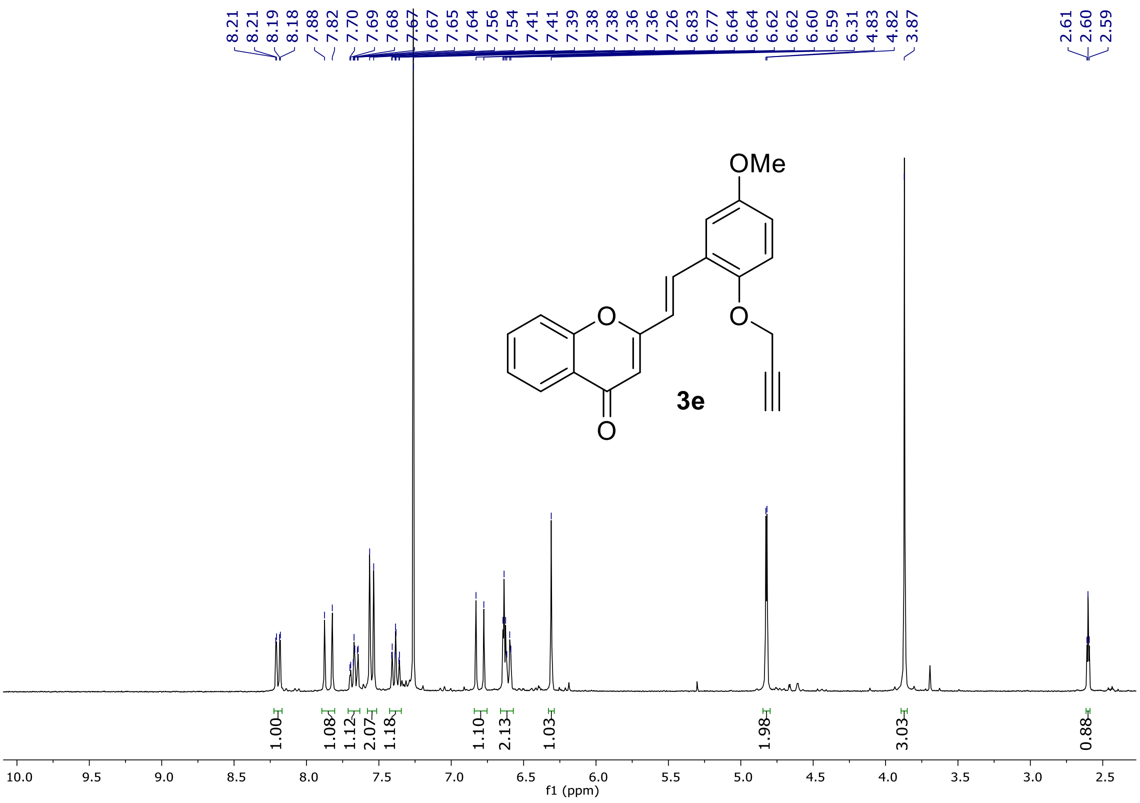

Figure S5. ^{1}H NMR spectrum of (*E*)-2-styrylchromone (**3e**) (300 Mhz, $CDCl_3$).

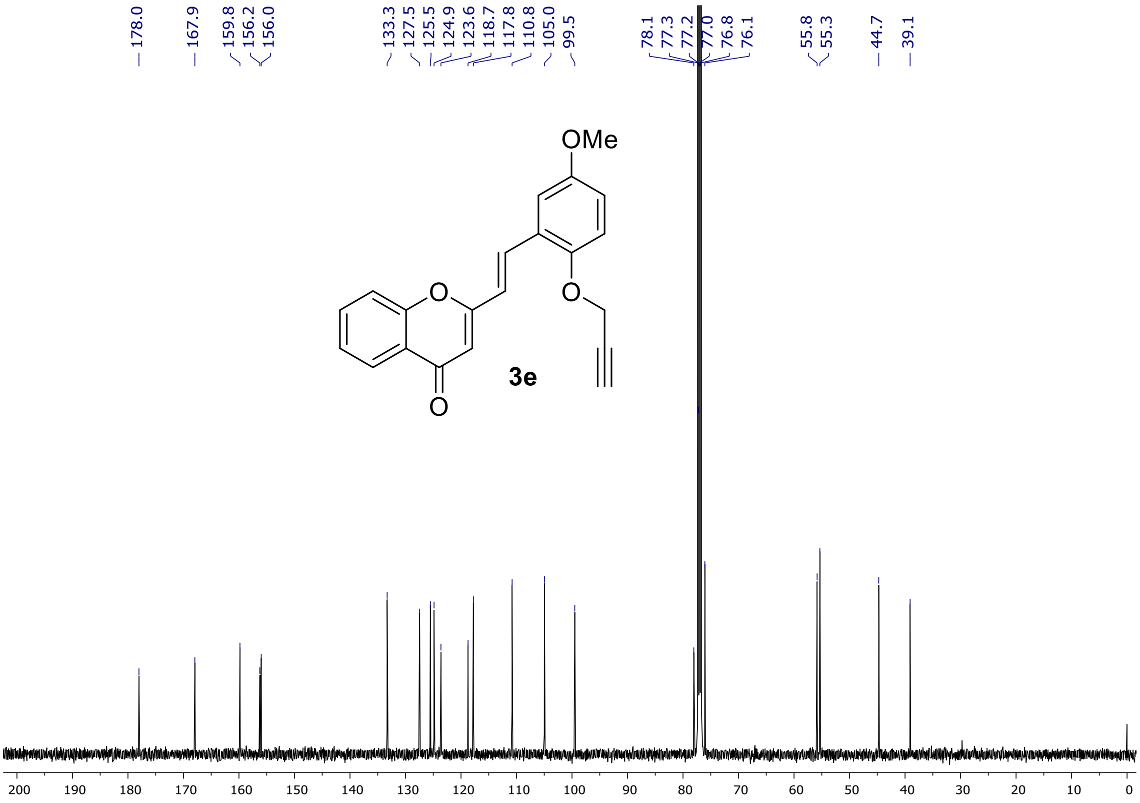

Figure S6. ^{13}C NMR spectrum of (*E*)-2-styrylchromone (**3e**) (125 Mhz, $CDCl_3$).

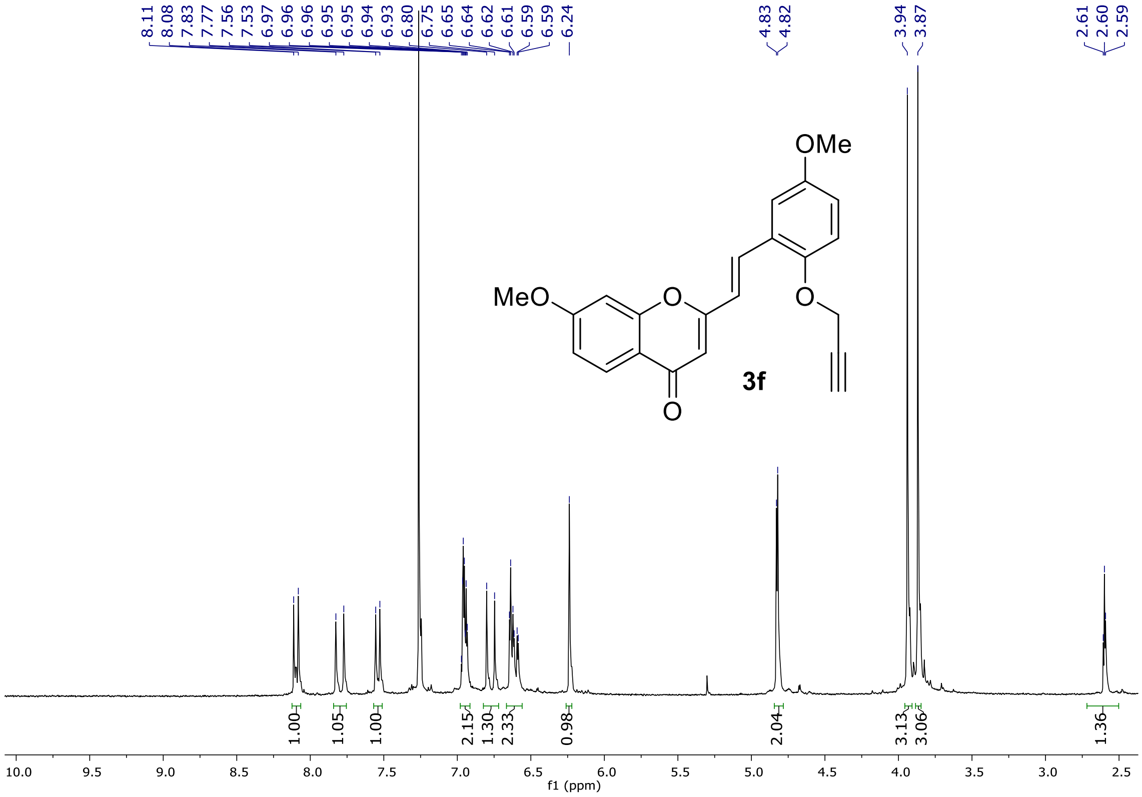

Figure S7. ^{1}H NMR spectrum of (*E*)-2-styrylchromone (**3f**) (300 Mhz, $CDCl_3$).

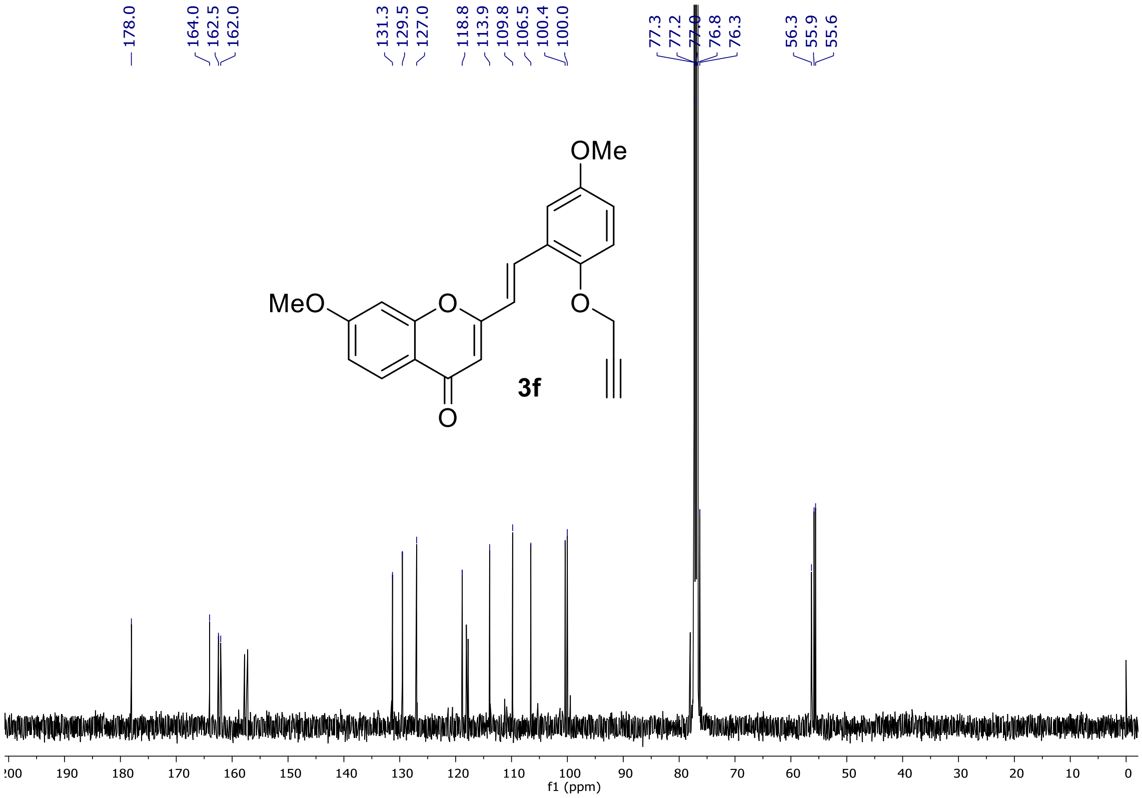

Figure S8. ^{13}C NMR spectrum of (*E*)-2-styrylchromone (**3f**) (75 Mhz, $CDCl_3$).

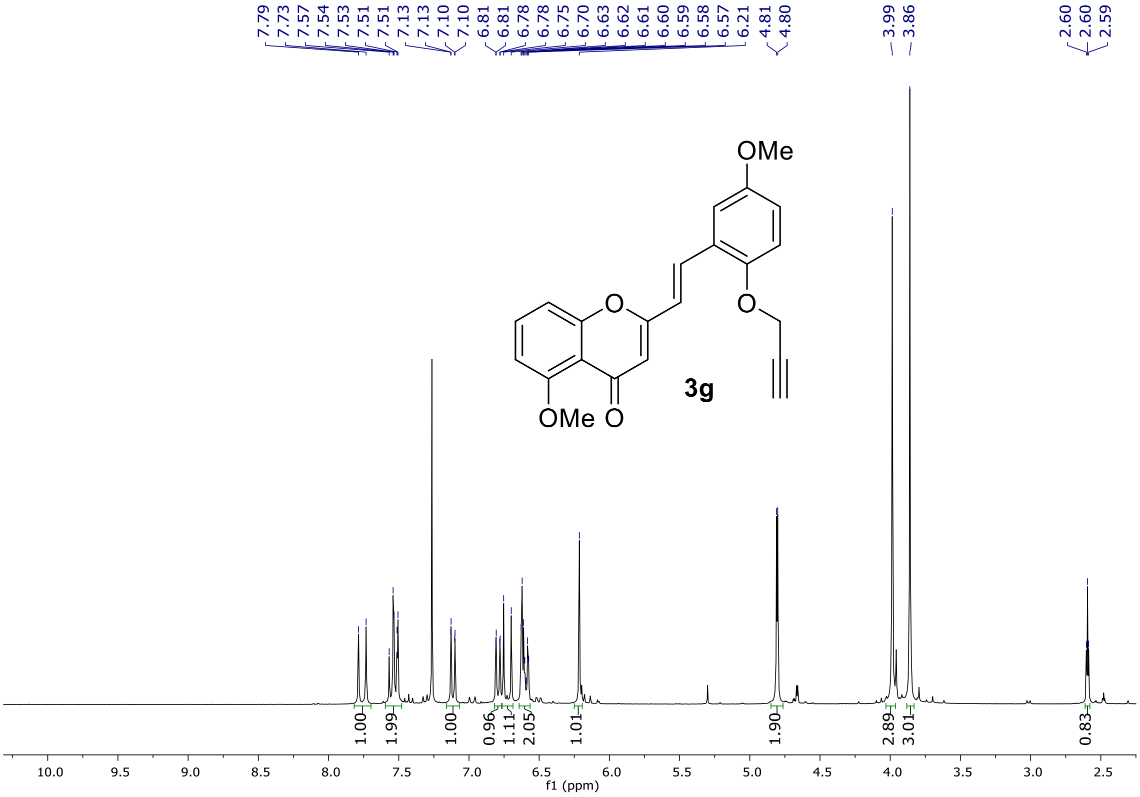

Figure S9. ^{1}H NMR spectrum of (*E*)-2-styrylchromone (**3g**) (300 Mhz, $CDCl_3$).

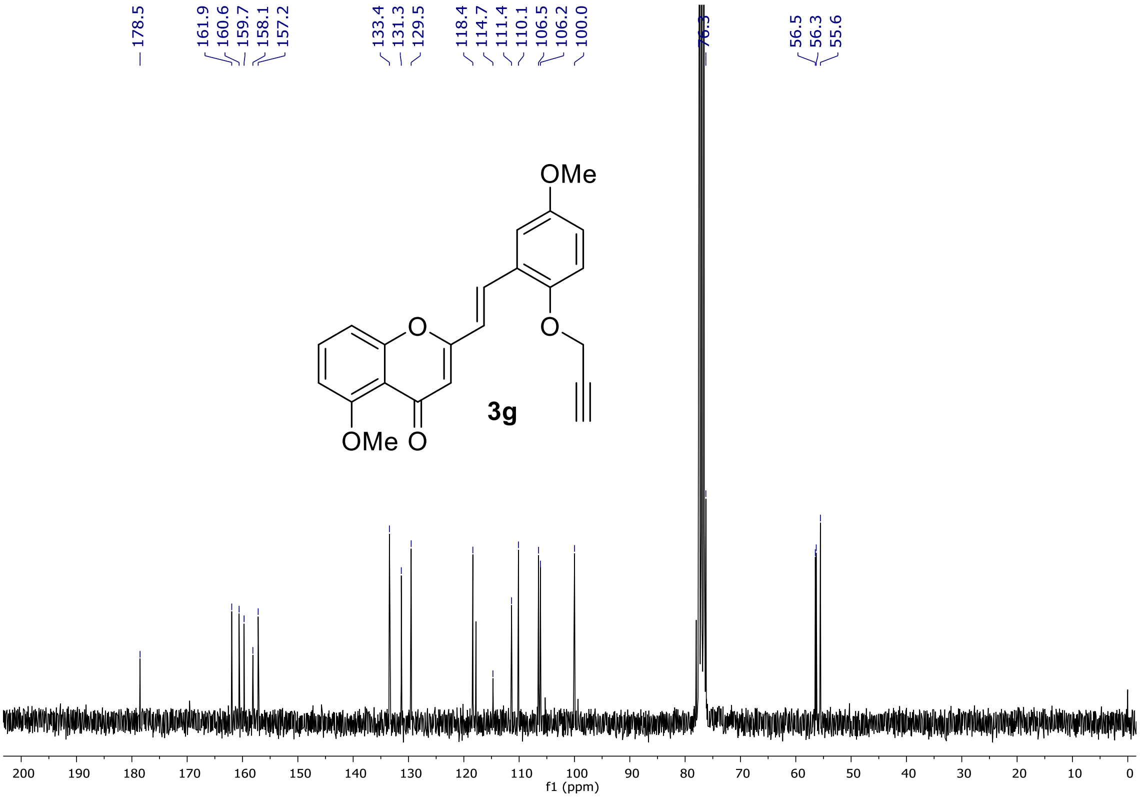

Figure S10. ^{13}C NMR spectrum of (*E*)-2-styrylchromone (**3g**) (75 Mhz, $CDCl_3$).

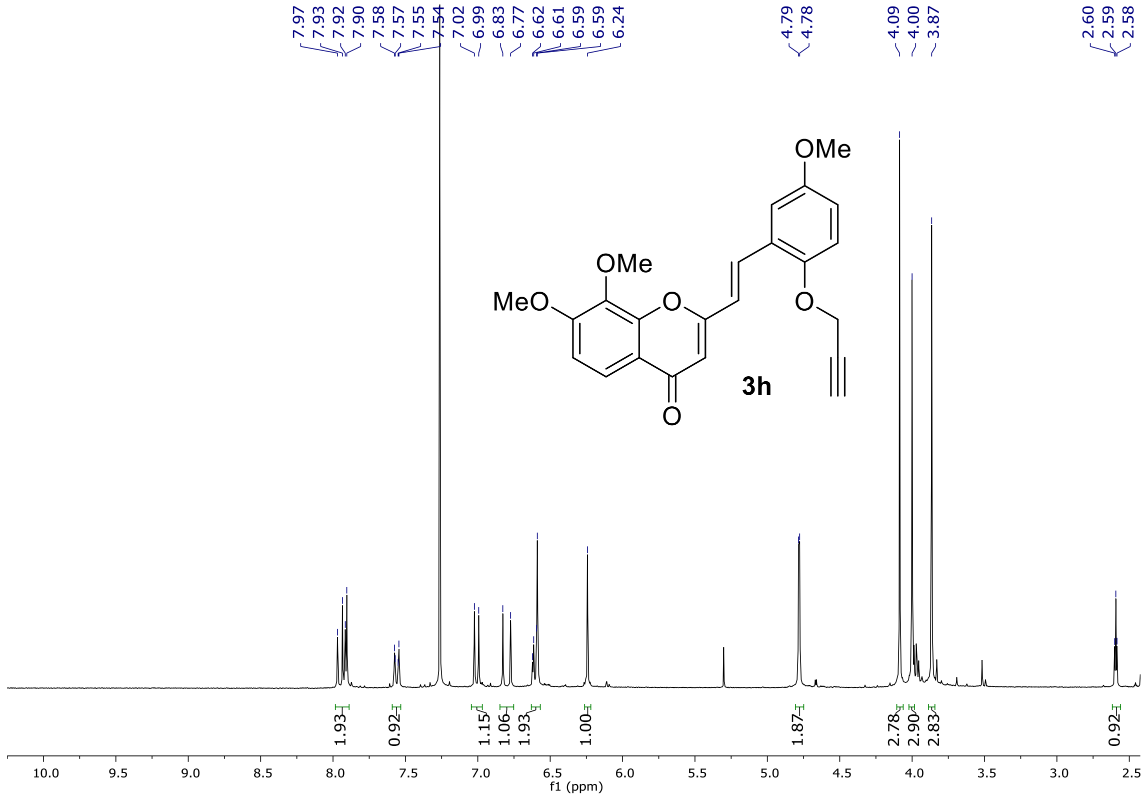

Figure S11. ^{1}H NMR spectrum of (*E*)-2-styrylchromone (**3g**) (300 Mhz, $CDCl_3$).

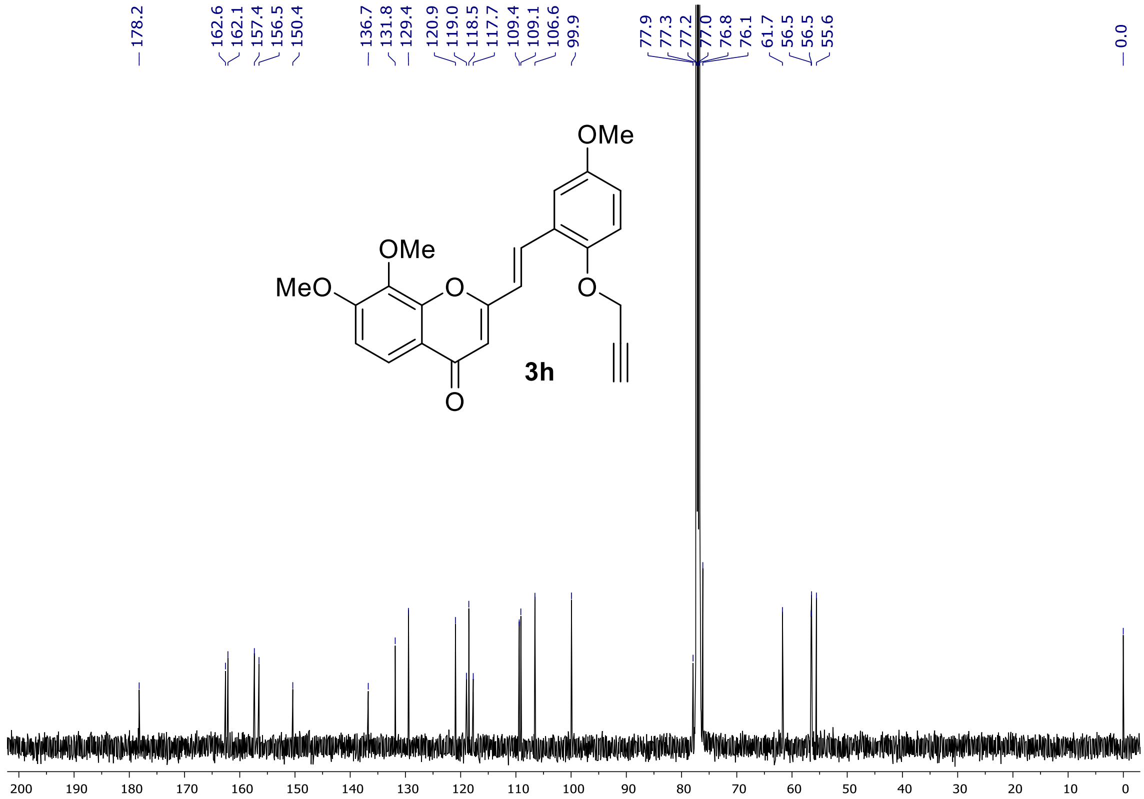

Figure S12. ^{13}C NMR spectrum of (*E*)-2-styrylchromone (**3h**) (125 Mhz, $CDCl_3$).

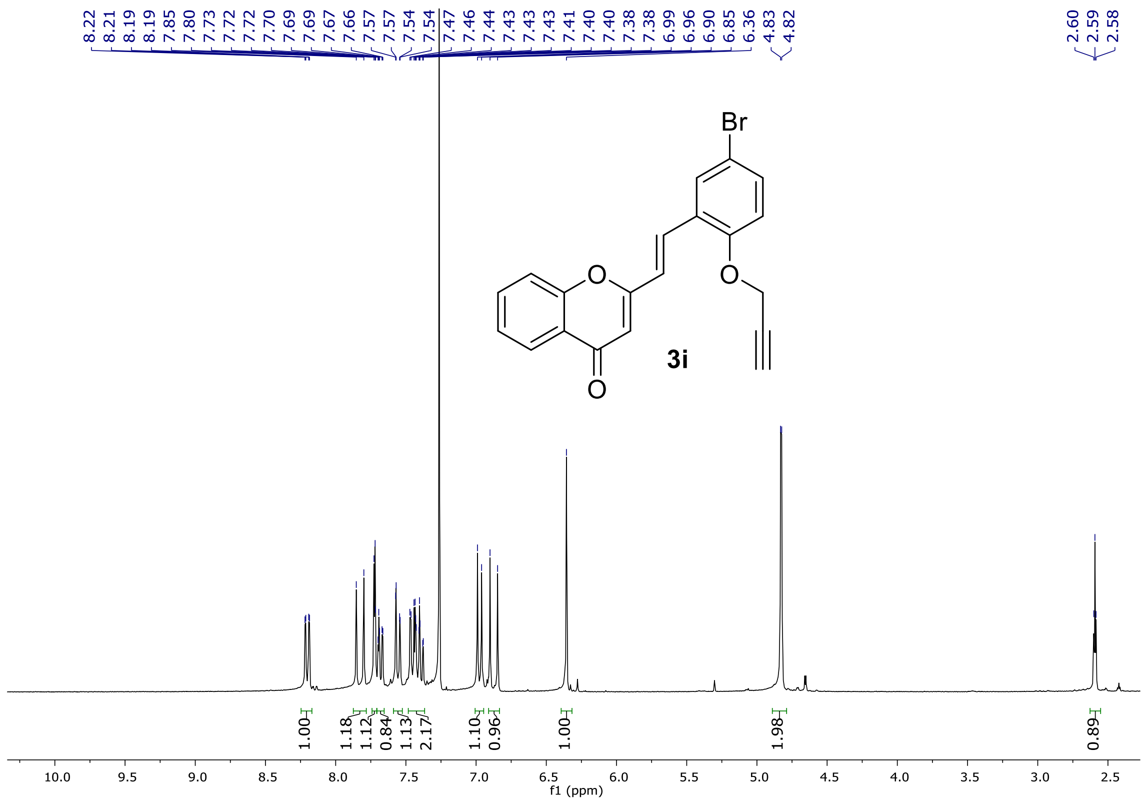

Figure S13. ^{1}H NMR spectrum of (*E*)-2-styrylchromone (**3i**) (300 Mhz, $CDCl_3$).

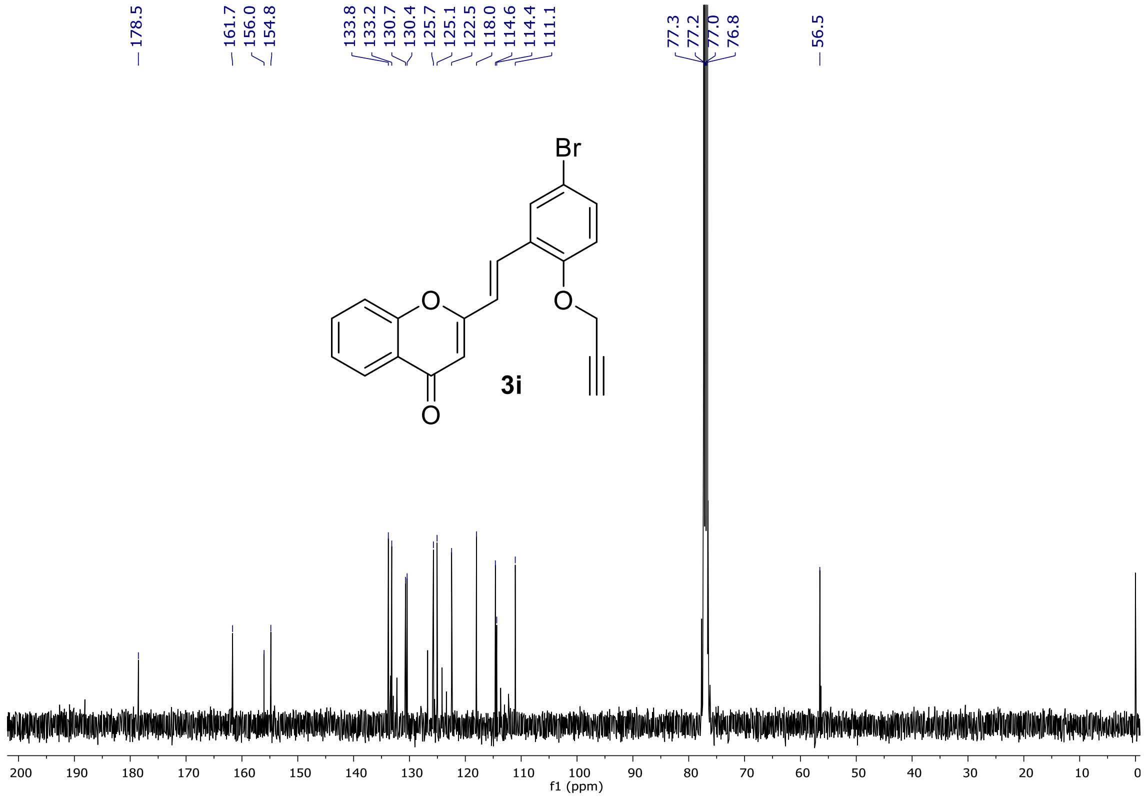

Figure S14. ^{13}C NMR spectrum of (*E*)-2-styrylchromone (**3i**) (75 Mhz, $CDCl_3$).

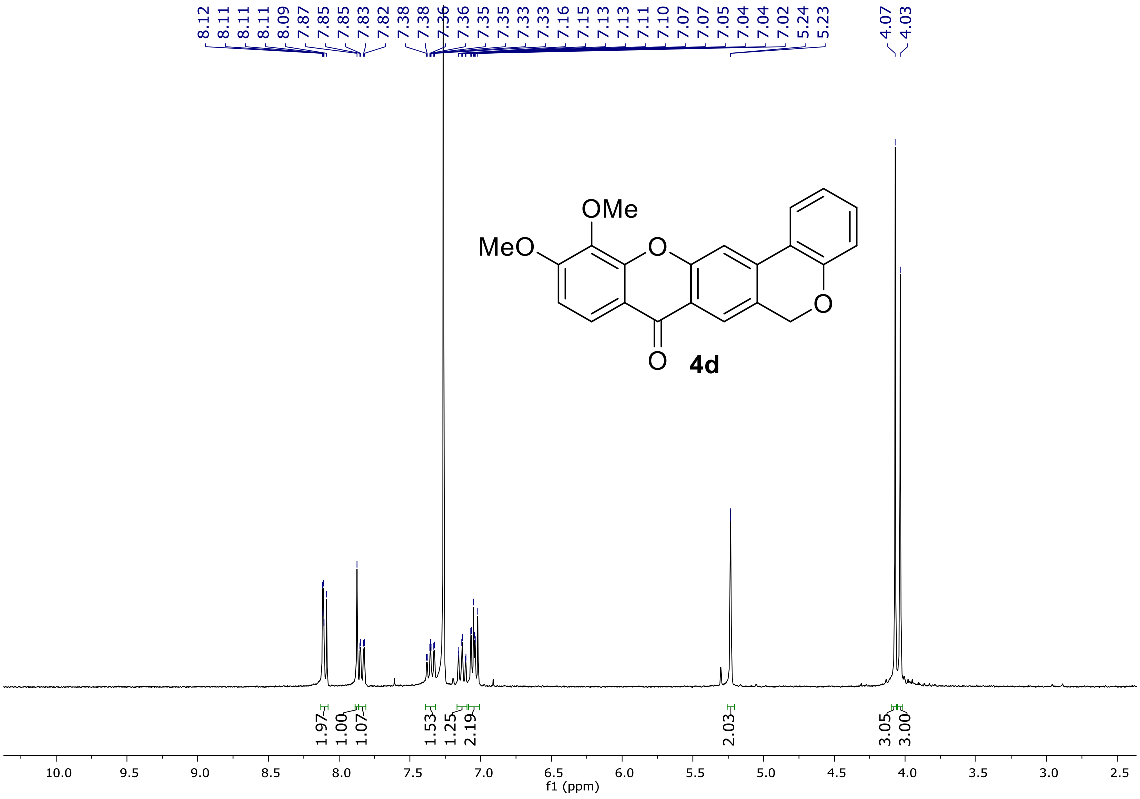

Figure S15. ^{1}H NMR spectrum of chromeno[3,4-*b*]xanthone (**4d**) (300 Mhz, $CDCl_3$).

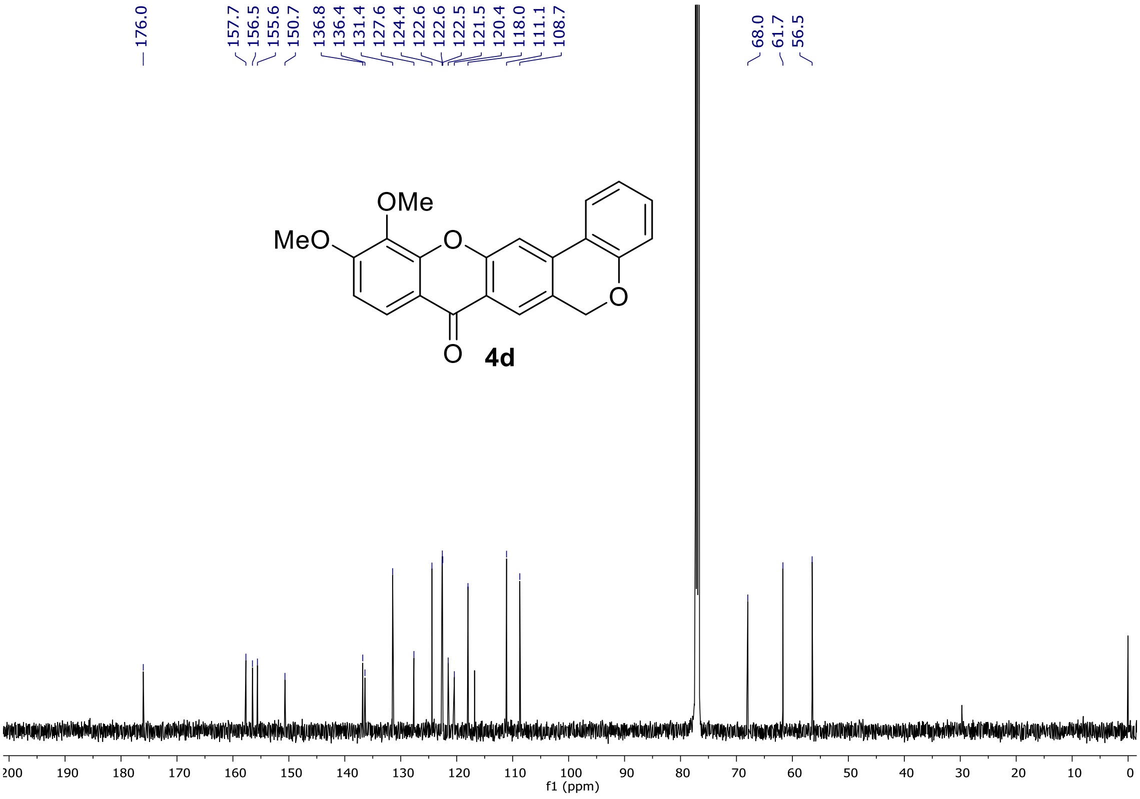

Figure S16. ^{13}C NMR spectrum of chromeno[3,4-*b*]xanthone (**4d**) (75 Mhz, $CDCl_3$).

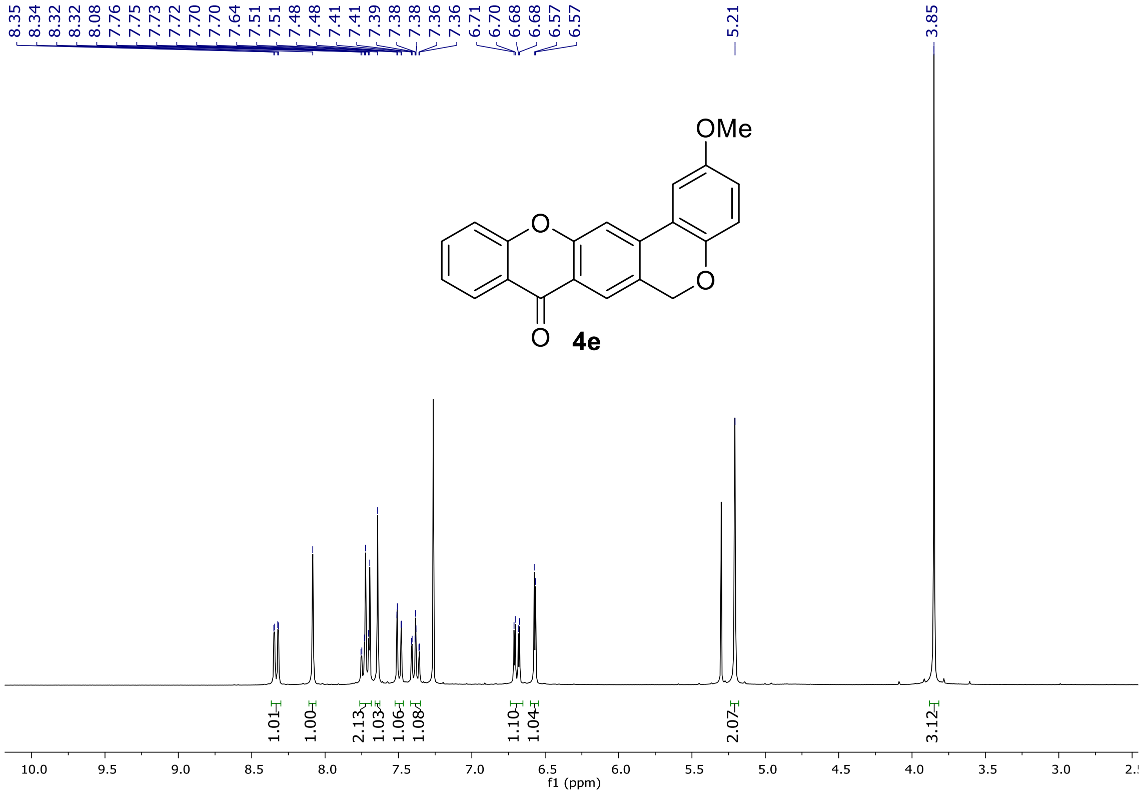

Figure S17. ^{1}H NMR spectrum of chromeno[3,4-*b*]xanthone (**4e**) (300 Mhz, $CDCl_3$).

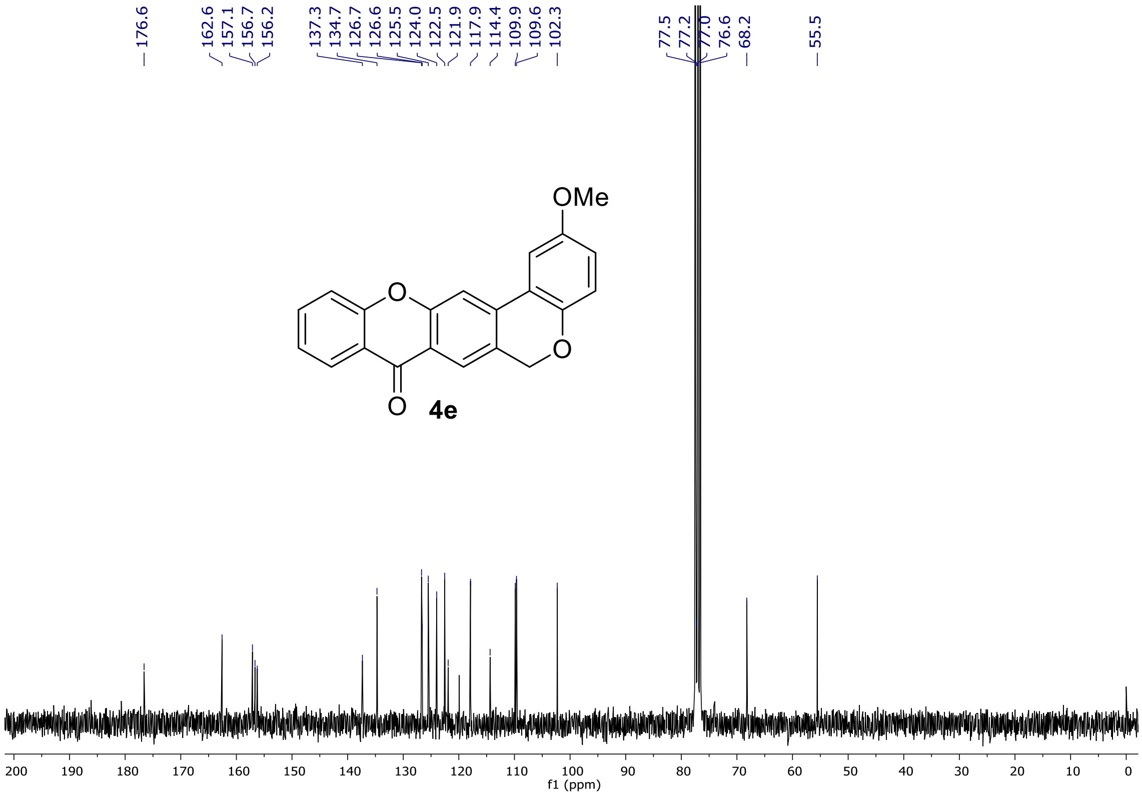

Figure S18. ^{13}C NMR spectrum of chromeno[3,4-*b*]xanthone (**4e**) (75 Mhz, $CDCl_3$).

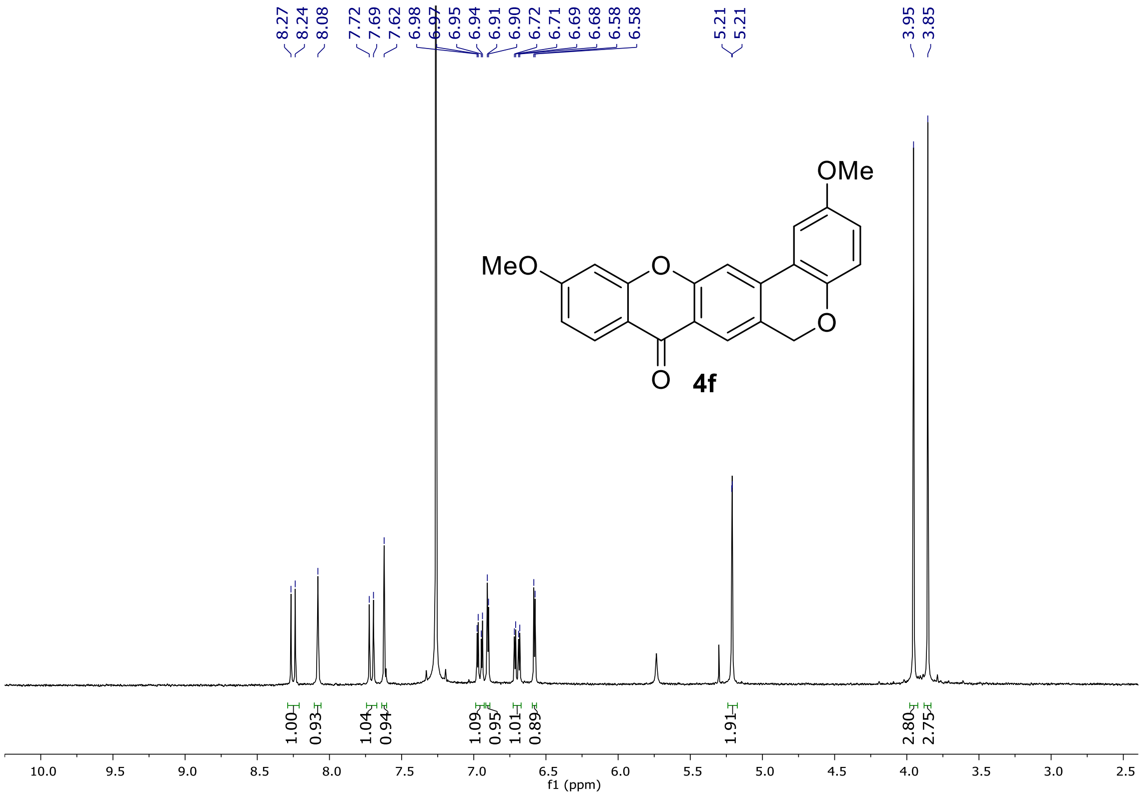

Figure S19. ^{1}H NMR spectrum of chromeno[3,4-*b*]xanthone (**4f**) (300 Mhz, $CDCl_3$).

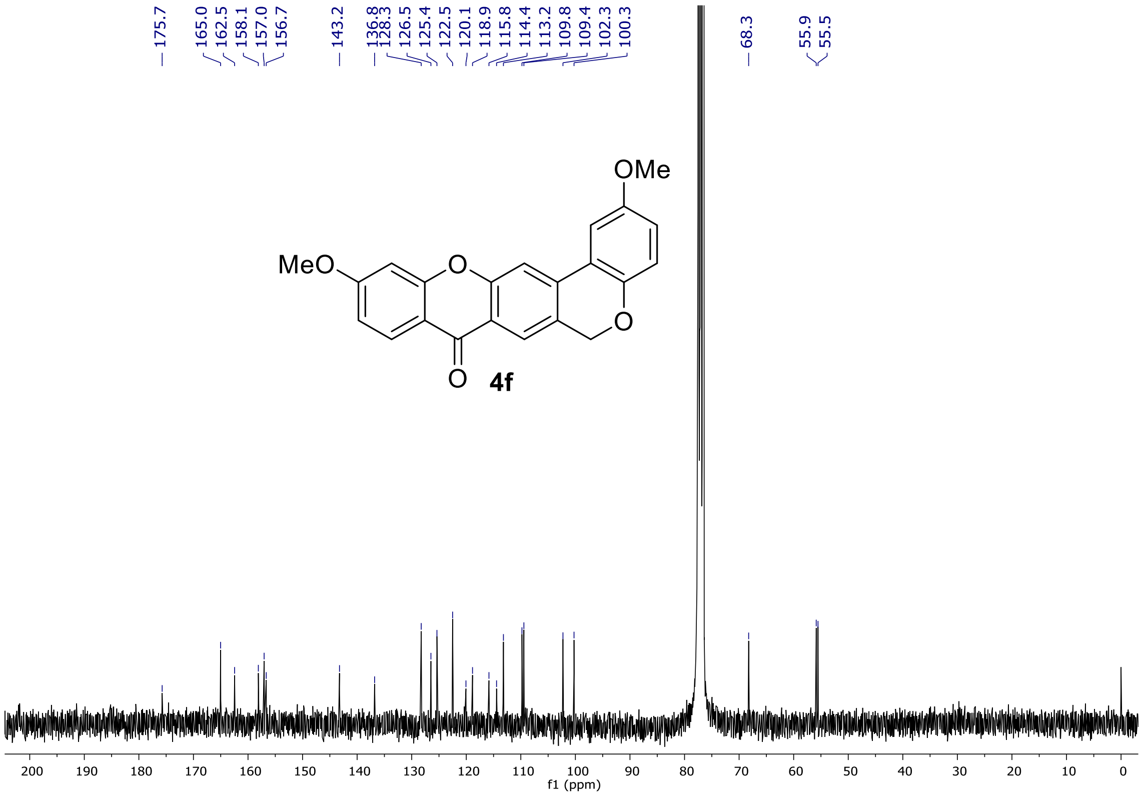

Figure S20. ^{13}C NMR spectrum of chromeno[3,4-*b*]xanthone (**4f**) (75 Mhz, $CDCl_3$).

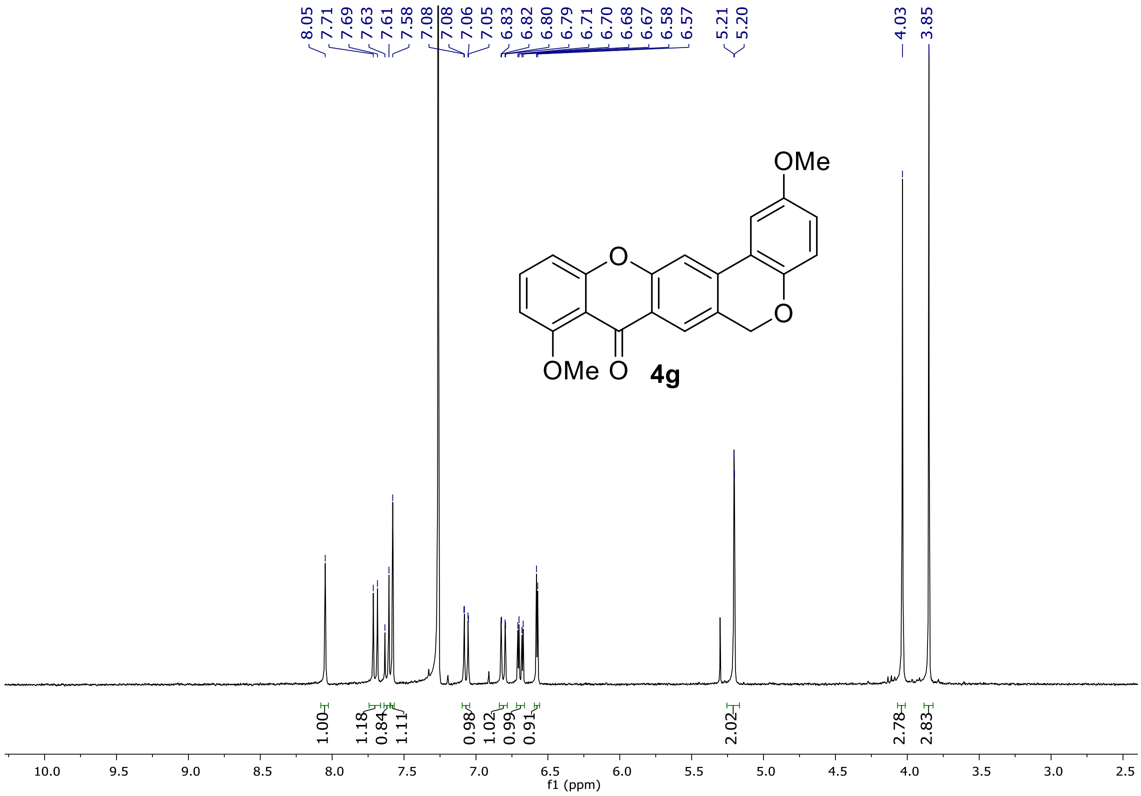

Figure S21. ^{1}H NMR spectrum of chromeno[3,4-*b*]xanthone (**4g**) (300 Mhz, $CDCl_3$).

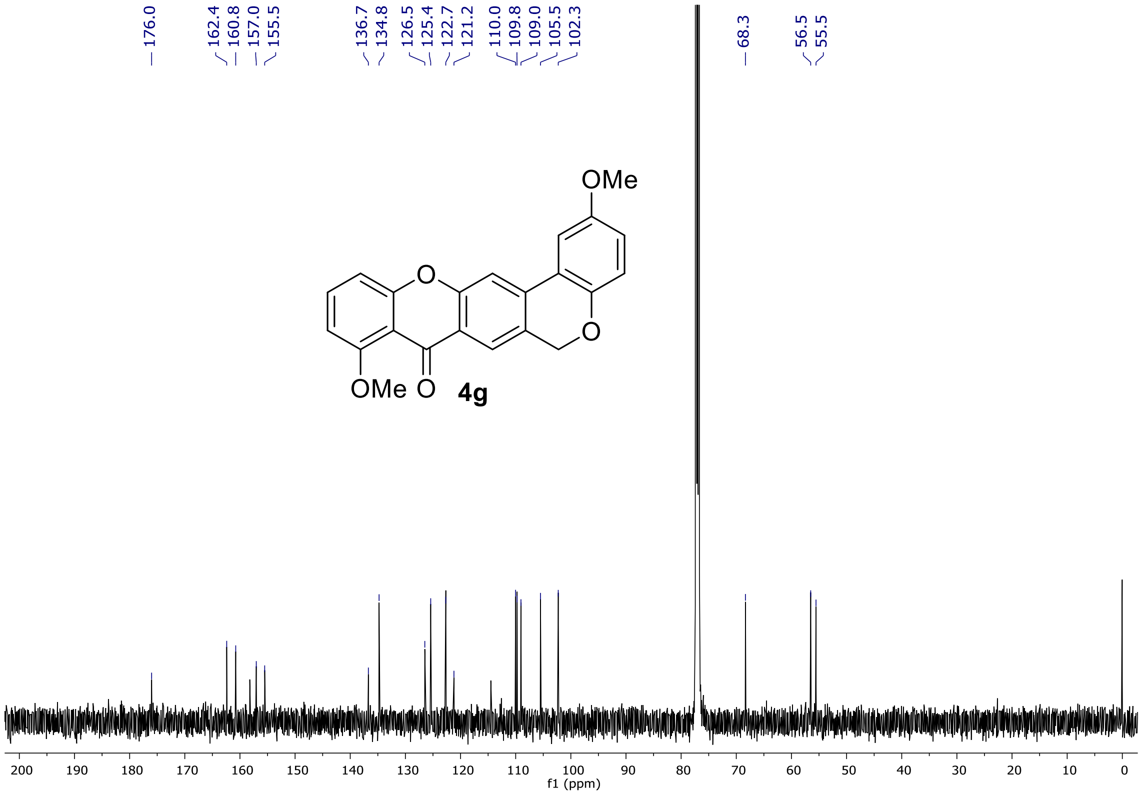

Figure S22. ^{13}C NMR spectrum of chromeno[3,4-*b*]xanthone (**4g**) (75 Mhz, $CDCl_3$).

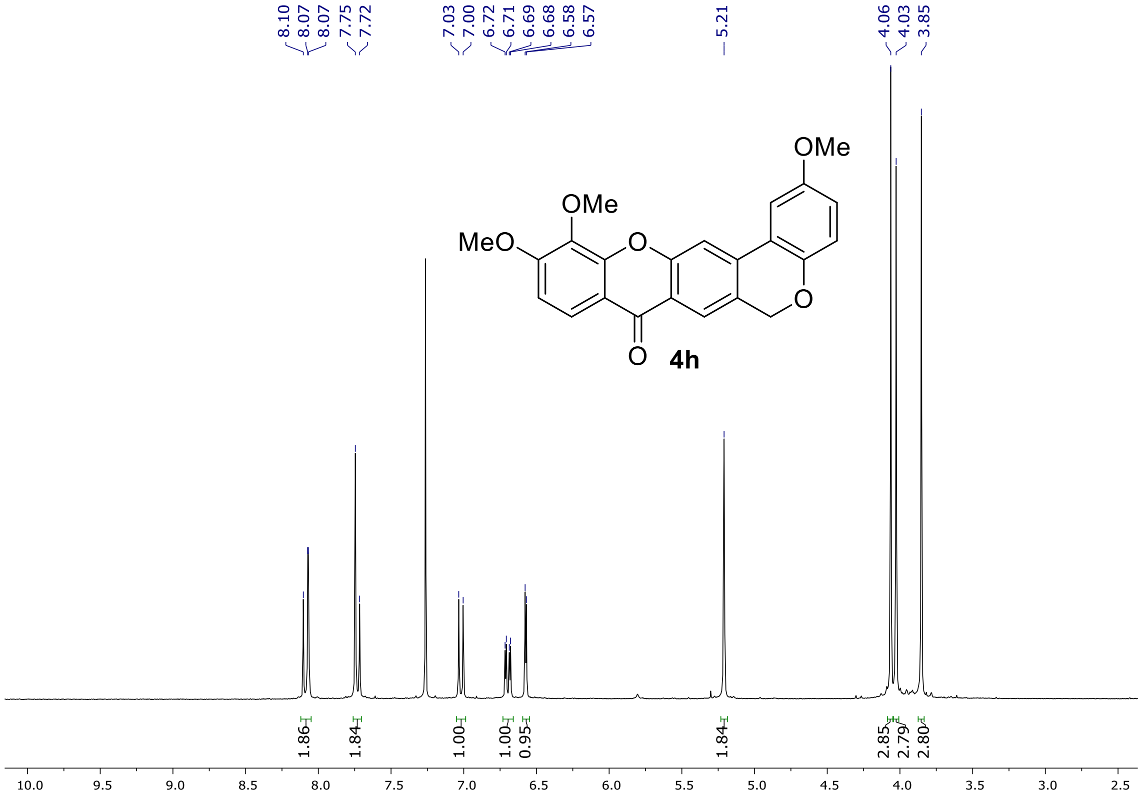

Figure S23. ^{1}H NMR spectrum of chromeno[3,4-*b*]xanthone (**4h**) (300 Mhz, $CDCl_3$).

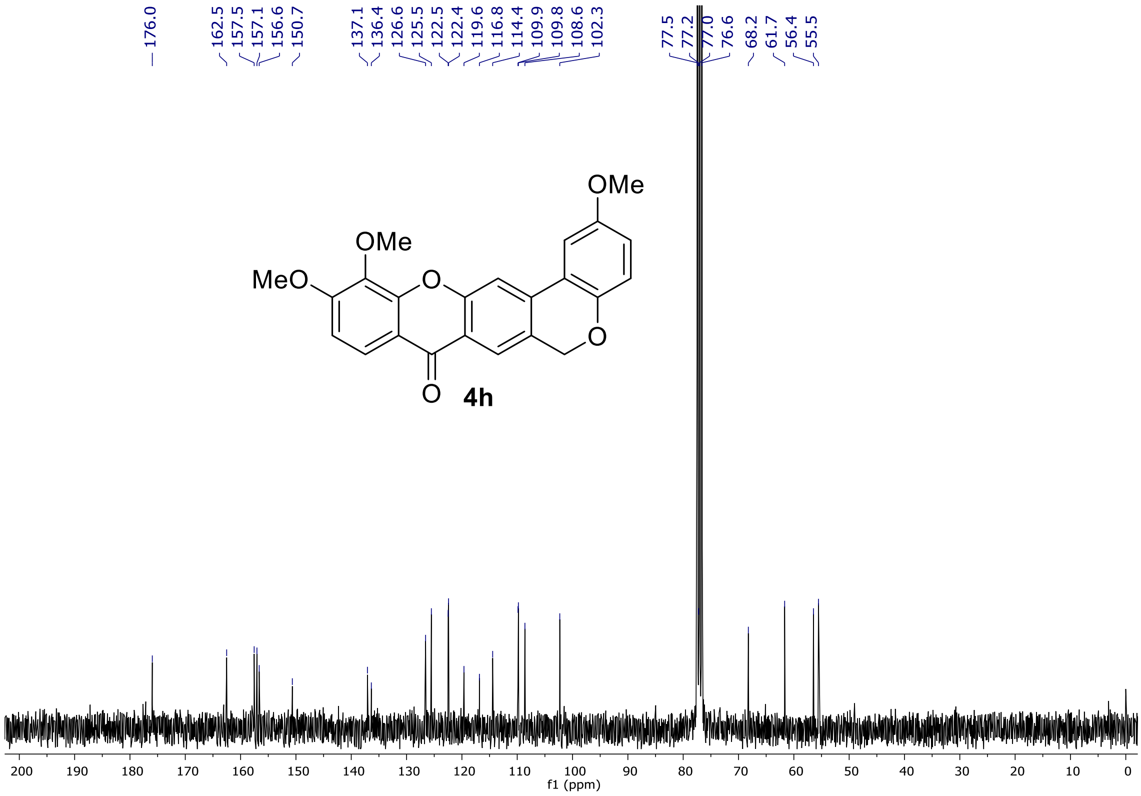

Figure S24. ^{13}C NMR spectrum of chromeno[3,4-*b*]xanthone (**4h**) (75 Mhz, $CDCl_3$).

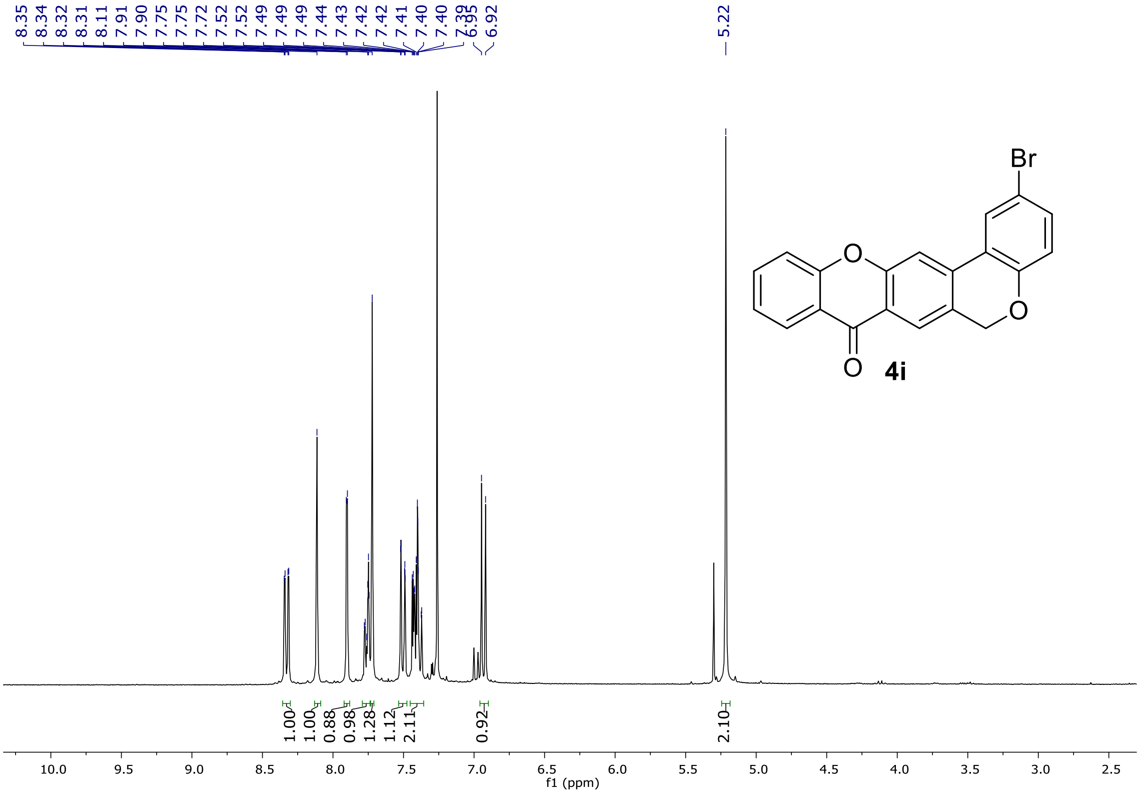

Figure S25. ^{1}H NMR spectrum of chromeno[3,4-*b*]xanthone (**4i**) (300 Mhz, $CDCl_3$).

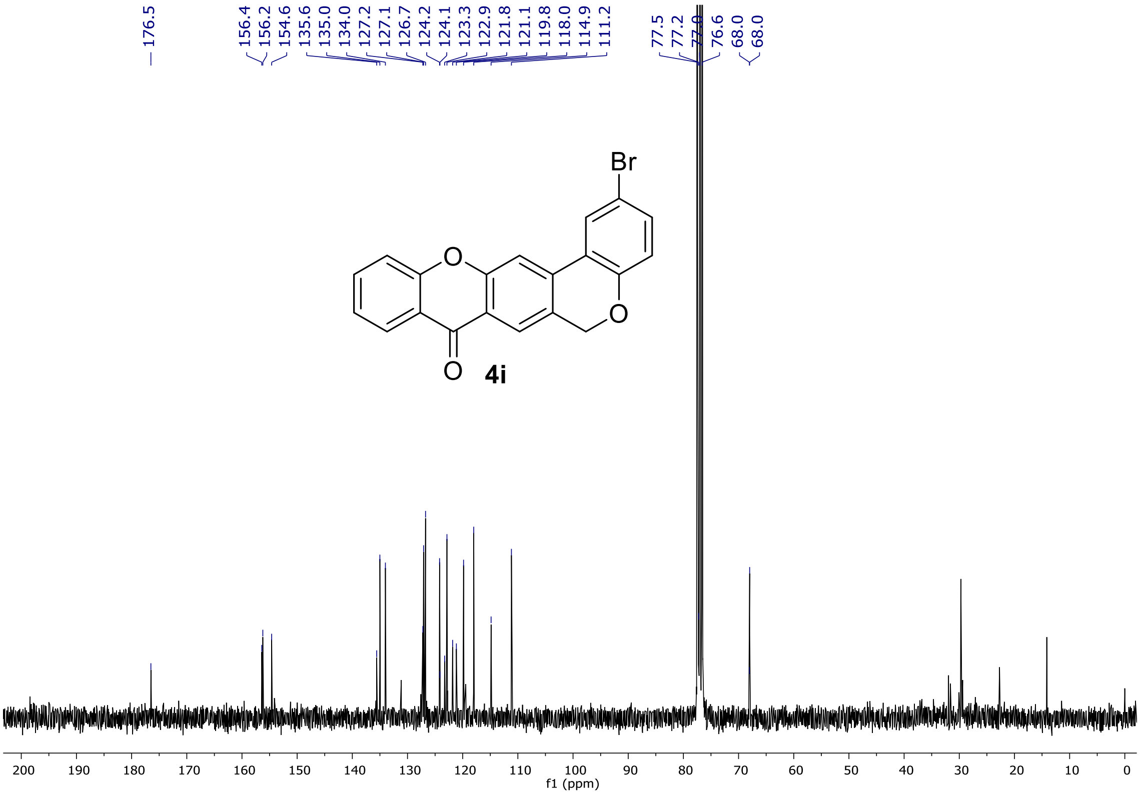

Figure S26. ^{13}C NMR spectrum of chromeno[3,4-*b*]xanthone (**4i**) (75 Mhz, $CDCl_3$).

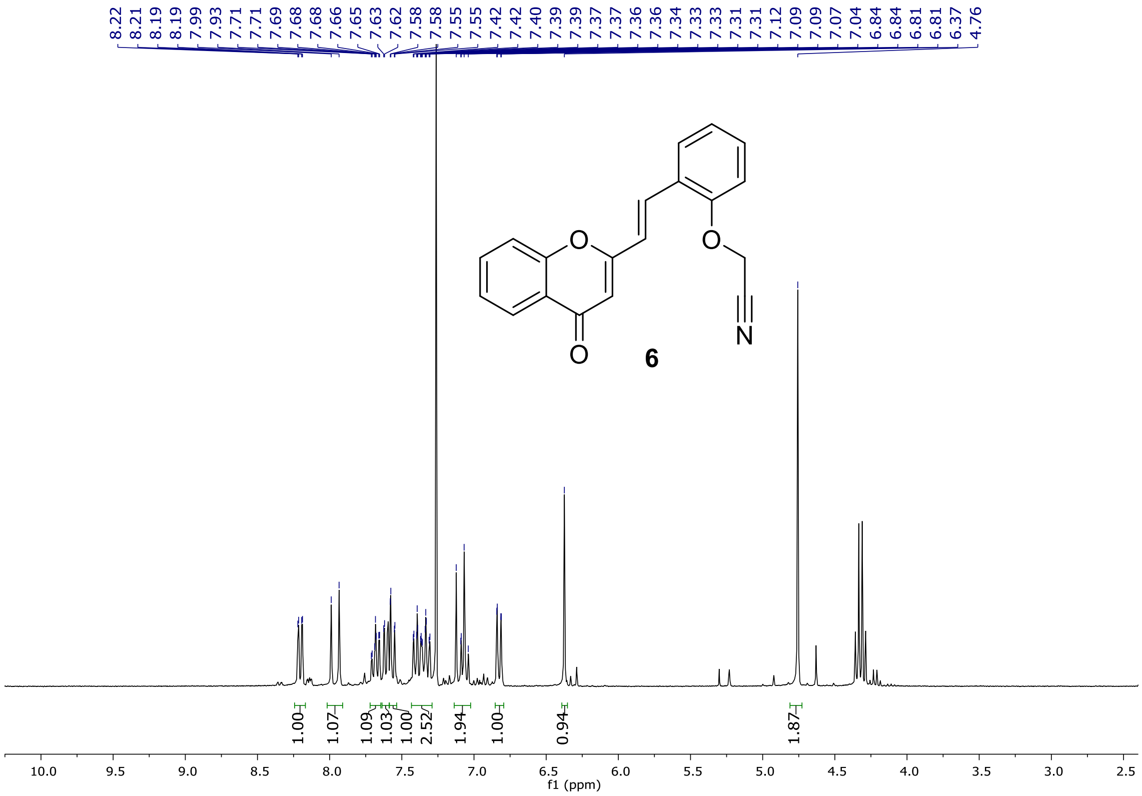

Figure S27. ^{1}H NMR spectrum of (*E*)-2-styrylchromone (**6**) (300 Mhz, $CDCl_3$).

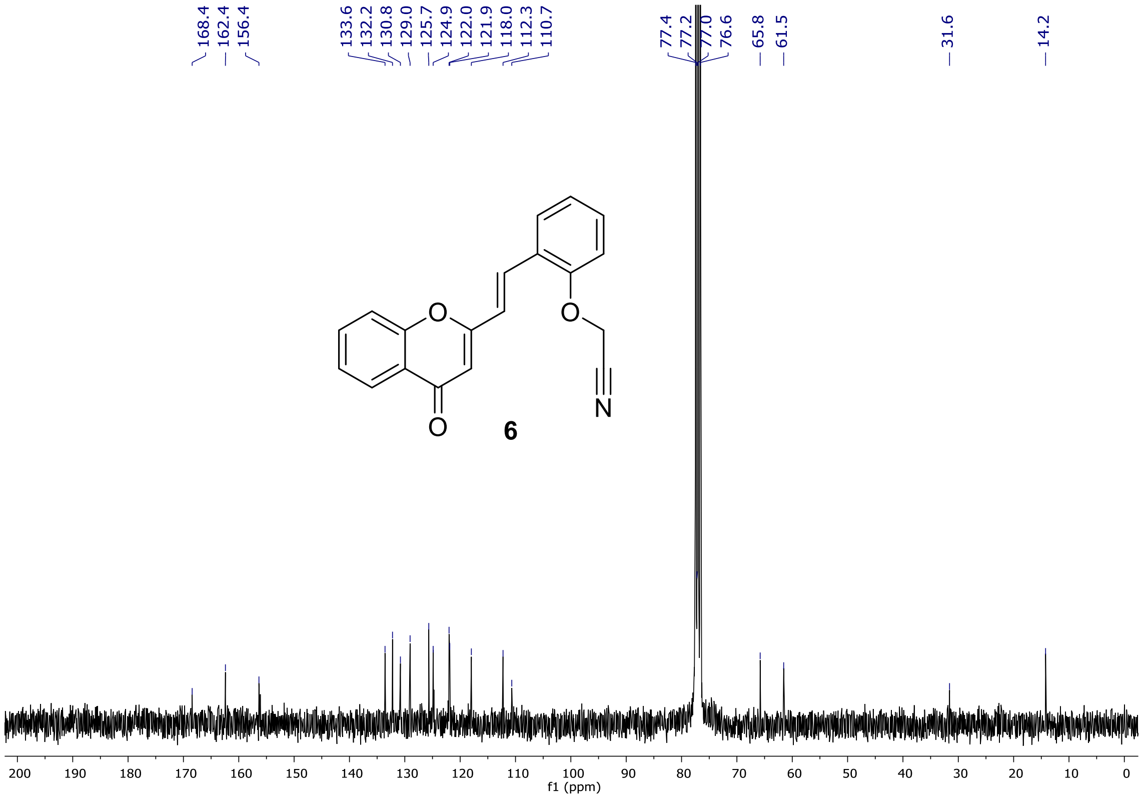

Figure S28. ^{13}C NMR spectrum of (*E*)-2-styrylchromone (**6**) (75 Mhz, $CDCl_3$).

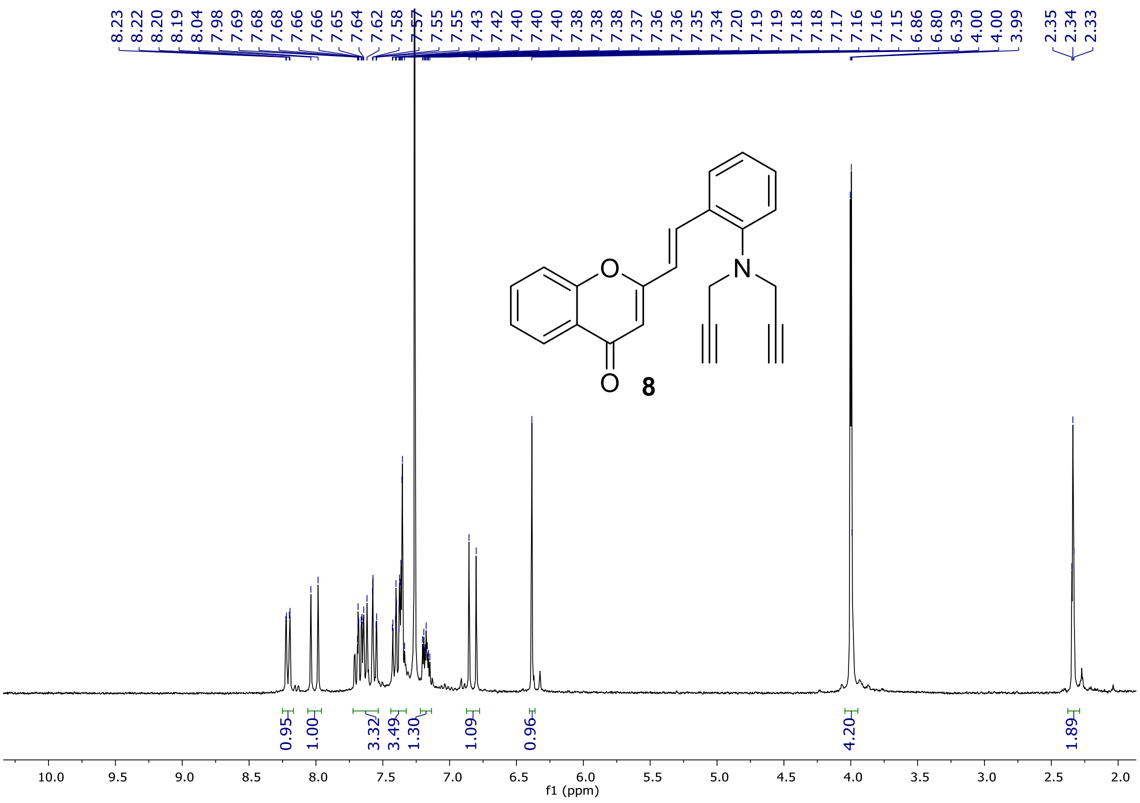

Figure S29. ^{1}H NMR spectrum of (*E*)-2-styrylchromone (**8**) (300 Mhz, $CDCl_3$).

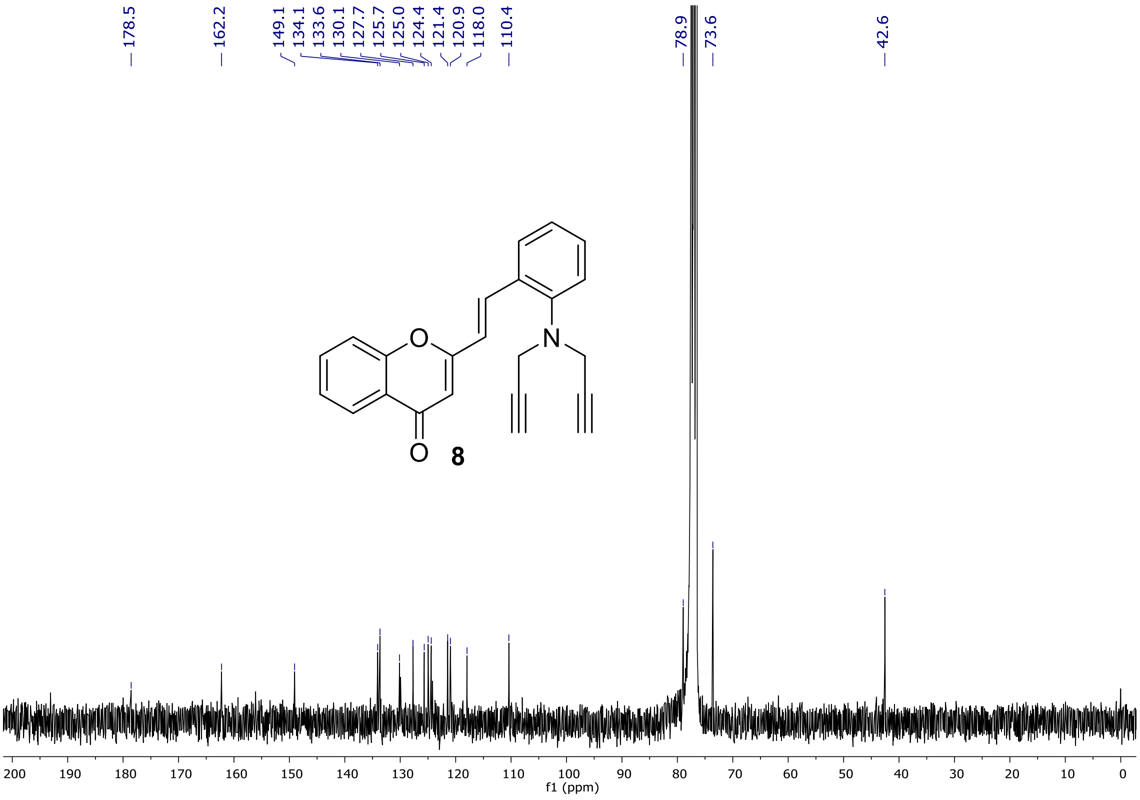

Figure S30. ^{13}C NMR spectrum of (*E*)-2-styrylchromone (**8**) (75 Mhz, $CDCl_3$).

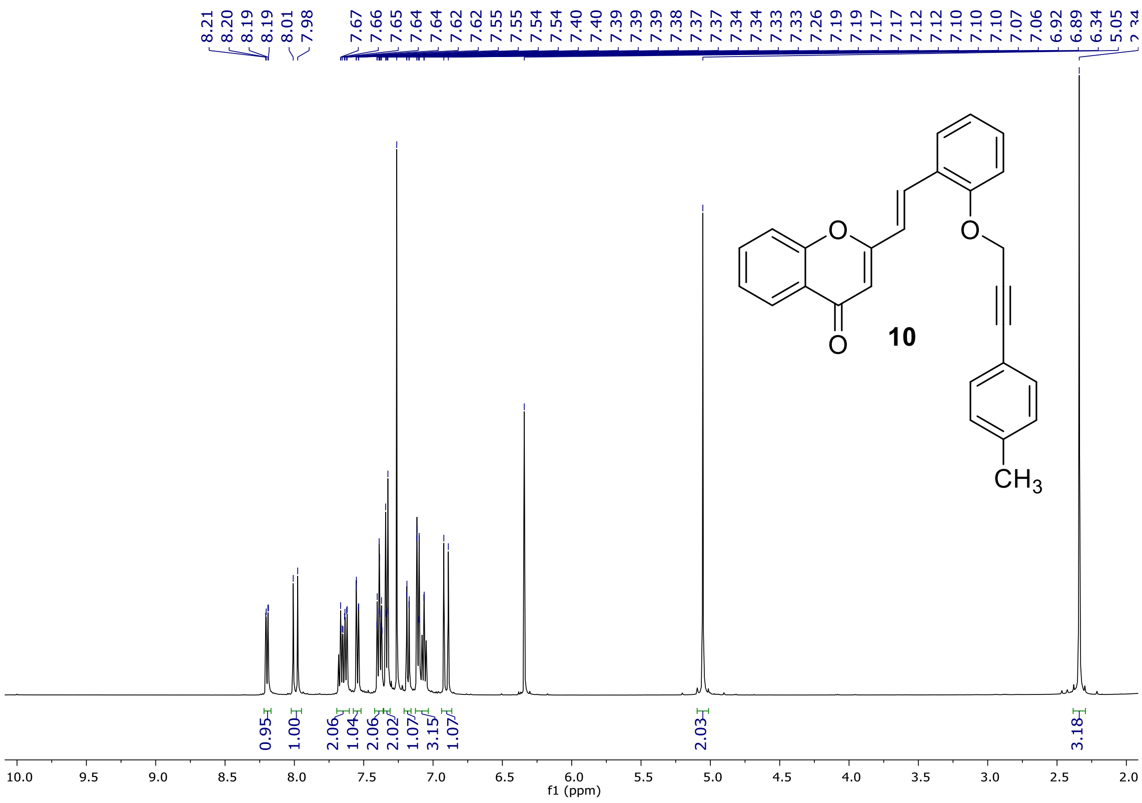

Figure S31. ^{1}H NMR spectrum of (*E*)-2-styrylchromone (**10**) (500 Mhz, $CDCl_3$).

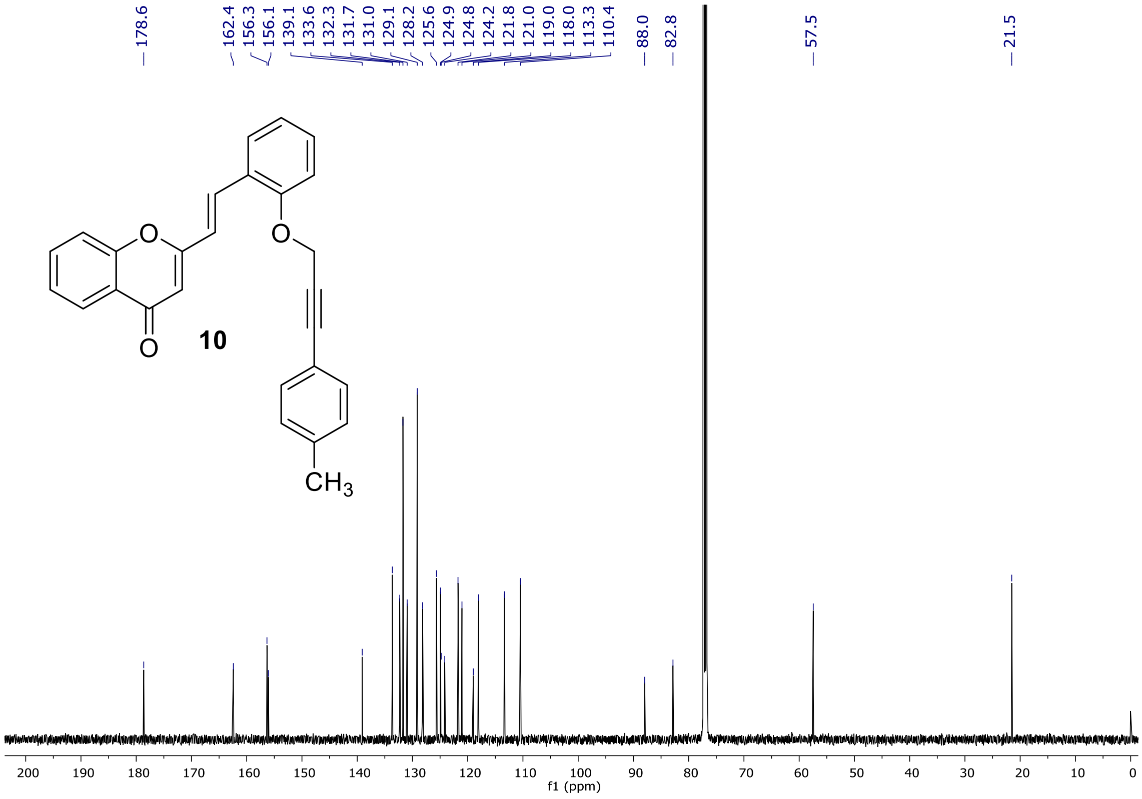

Figure S32. ^{13}C NMR spectrum of (*E*)-2-styrylchromone (**10**) (125 Mhz, $CDCl_3$).

References:

1. Shaw, A. Y.; Chang, C.-Y.; Liau, H.-H.; Lu, P.-J.; Chen, H.-L.; Yang, C.-N.; Li, H.-Y., Synthesis of 2-styrylchromones as a novel class of antiproliferative agents targeting carcinoma cells. *Eur. J. Med. Chem.* **2009,** *44*, 2552-2562.
2. Hoplamaz, E.; Keskin, S.; Balci, M., Regioselective Synthesis of Benzo[h][1,6]-naphthyridines and Chromenopyrazinones through Alkyne Cyclization. *Eur. J. Org. Chem.* **2017,** 1489-1497.
